# Peptide Backbone Directed Self‐Assembly of Merocyanine Oligomers into Duplex Structures

**DOI:** 10.1002/anie.202200120

**Published:** 2022-03-23

**Authors:** Bin Liu, Yvonne Vonhausen, Alexander Schulz, Claudia Höbartner, Frank Würthner

**Affiliations:** ^1^ Institut für Organische Chemie Universität Würzburg Am Hubland 97074 Würzburg Germany; ^2^ Center for Nanosystems Chemistry (CNC) Universität Würzburg Theodor-Boveri-Weg 97074 Würzburg Germany

**Keywords:** Dipole-Dipole Interaction, Duplex Structure, Dye Assembly, Merocyanine, Peptide Backbone

## Abstract

The pseudopeptide backbone provided by *N*‐(2‐aminoethyl)‐glycine oligomers with attached nucleobases has been widely utilized in peptide nucleic acids (PNAs) as DNA mimics. Here we demonstrate the suitability of this backbone for the formation of structurally defined dye stacks. Toward this goal a series of peptide merocyanine (PMC) dye oligomers connected to a *N*‐(2‐aminoethyl)‐glycine backbone were prepared through peptide synthesis. Our concentration‐, temperature‐ and solvent‐dependent UV/Vis absorption studies show that under the control of dipole–dipole interactions, smaller‐sized oligomers consisting of one, two or three dyes self‐assemble into defined duplex structures containing two up to six chromophores. In contrast, upon further extension of the oligomer, the chosen peptide backbone cannot direct the formation of a defined duplex architecture anymore due to intramolecular aggregation between the dyes. For all aggregate species a moderate aggregation‐induced emission enhancement is observed.

## Graphical abstract

Self‐assembly of peptide backbone connected merocyanine oligomers leads to the formation of DNA‐like duplex structures containing two, four, and six chromophores. Whether self‐assembly or folding is preferred depends on the length of the oligomers, as well as the concentration and polarity of the solvents.

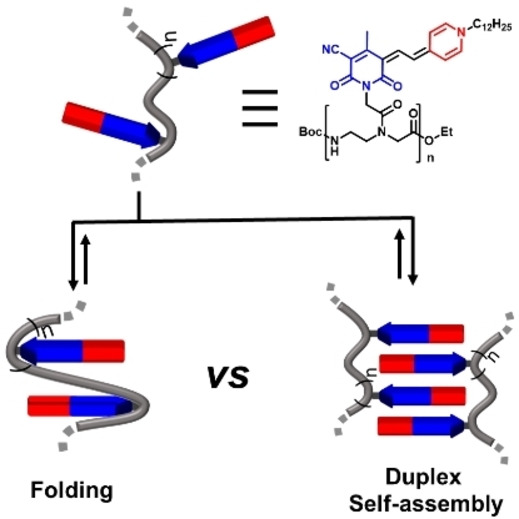

Dye assembly has attracted considerable attention in the past two decades.^[1]^ This is not only because well‐ordered ensembles of multiple chromophores afford unique and desirable photophysical and photochemical properties different from those of the monomers but also because the functional properties of π‐conjugated molecular solid‐state materials are governed by the electronic coupling between the molecular building blocks.[^2^, ^3^] To explore structural and functional properties, multichromophore systems with well‐defined size and structure are needed.[^1^, ^4^] So far, chemists have developed a variety of strategies to construct multichromophore assemblies which include steric shielding,[^5^, ^6^] templating,[^7^, ^8^] and backbone‐directed self‐assembly.[^9^, ^10^, ^11^] These examples reveal general strategies for the design of dimers and tetramers but also highlight the lack of a general approach towards larger oligomers of defined structure and size. In contrast, the well‐ordered double helix structure of DNA demonstrates a base pair guided backbone‐directed self‐assembly process that enables the formation of DNA double strands of any length. In principle, the sugar phosphate backbone found in DNA can also be utilized for the assembly of other π‐scaffolds which, however, limits the research to aqueous environments and π‐scaffolds often appended to natural nucleobases.[^12^, ^13^] In another approach, dyes are embedded within DNA duplex structures by replacing entire nucleotides in the phosphodiester backbone, but here it is challenging to accommodate the dyes within the DNA backbone in a predictable supramolecular arrangement due to the spatial constraints of the DNA duplex.[^14^, ^15^, ^16^, ^17^] Inspired by research on peptide nucleic acids (PNAs)[^18^, ^19^] and research on the folding of peptide backbones driven by donor‐acceptor interactions[^20^, ^21^] we herein explore the suitability of *N*‐(2‐aminoethyl)‐glycine‐based pseudopeptide backbones for the formation of oligomeric dye duplex structures (Figure 1).

**Figure 1:**
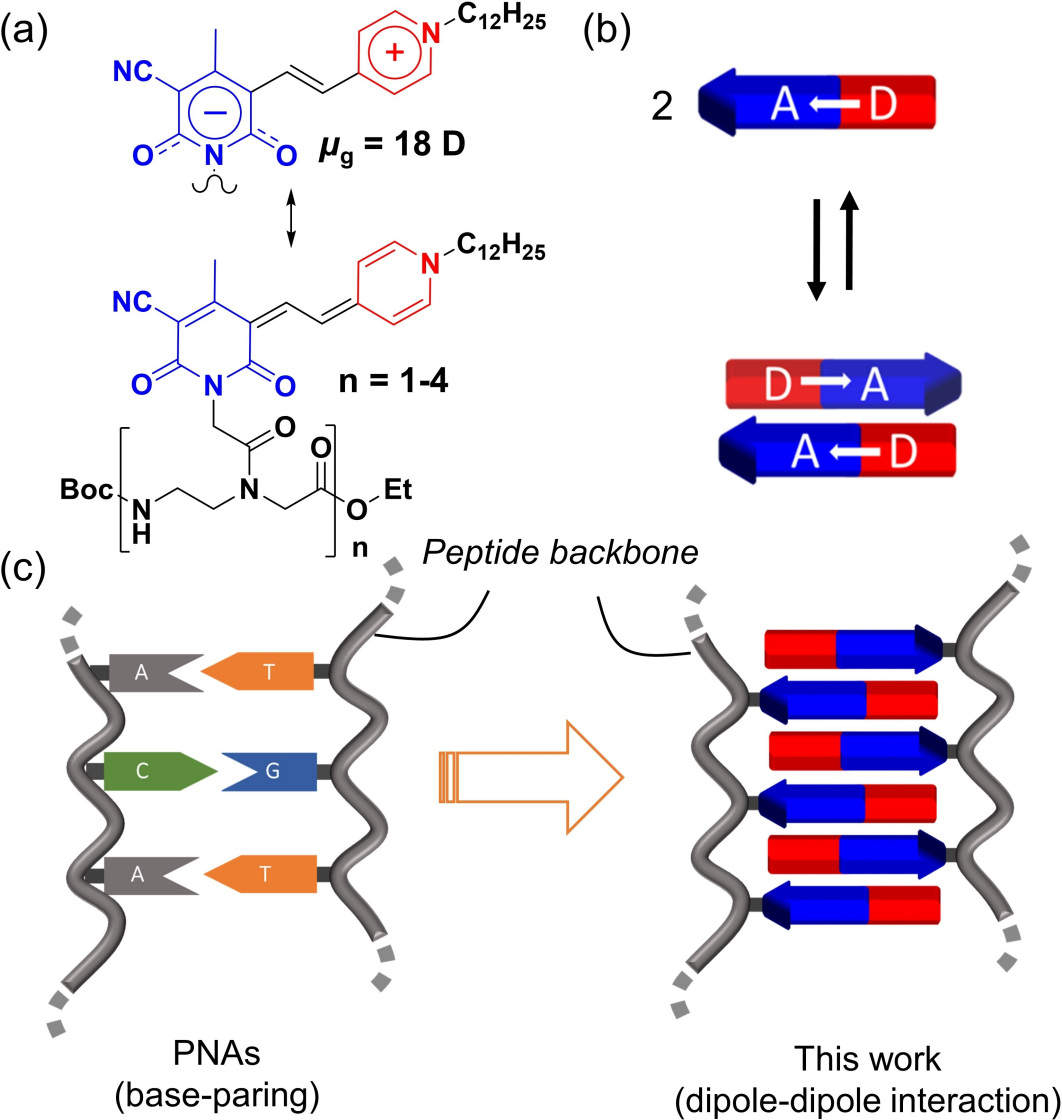
Concept for a peptide backbone‐directed self‐assembly of peptide MC (PMC) oligomers into duplex structures. a) Neutral and zwitterionic resonance structures of the merocyanine (MC) chromophore, and the chemical structures of the oligomers investigated in this study. b) Self‐assembly of dipolar MCs into antiparallel stacked dimers. c) Schematic representation of the self‐assembly of the complementary duplex of PNAs and peptide MC oligomers.

*N*‐(2‐aminoethyl)‐glycine‐based pseudopeptide backbones have indeed been used very successfully as a substitute for the (deoxy)ribose phosphate backbone in nucleic acids.[^22^, ^23^] Because of the similarity of the structures, PNA can also form complementary duplexes of high stability with DNA and RNA through base‐paring.^[24]^ By introducing cyanine dyes into PNA, a series of PNA‐FIT probes was successfully constructed and applied to the imaging of specific mRNA molecules.^[25]^ Therefore, if a chromophore is used to replace the nucleobase on PNA, it should be possible to direct the formation of duplexes consisting of multiple chromophores. Advantageously, the *N*‐(2‐aminoethyl)‐glycine‐based peptide backbone is not charged and quite hydrophobic, thereby not limiting the studies to polar solvents and water. Whilst we anticipate that different chromophores can be easily attached to the PNA skeleton by stepwise deprotection‐coupling protocols, thereby enabling a large variety of hetero dye stacks, in our initial study we focus on the formation of dye stacks consisting of only one dye, i.e. the dipolar merocyanine (MC) dye shown in Figure 1a.^[26]^ As shown in our earlier work the self‐assembly of this MC dye is strongly supported by electrostatic dipole‐dipole interactions (Figure 1b) due to a high ground state dipole moment (*μ*_g_=18 D) originating from a pronounced contribution of the zwitterionic resonance structure (Figure 1a).[^26^, ^27^] Therefore, the interaction between these MCs is directional, similar to the directionality of hydrogen bonds.^[28]^ Accordingly, similar to the directional base pairing that directs the self‐assembly of nucleic acid sequences in DNA, directional interactions of MC dyes should drive peptide backbone‐attached MC oligomers (Figure 1a) into ordered double‐stranded architectures under controlled conditions (Figure 1c).^[29]^

Peptide merocyanine (PMC) oligomers **1**_***n***_ were synthesized according to the route outlined in Scheme 1 (see the Supporting Information for details). Monomer **1** bearing protected amine and carboxylic acid functional groups was obtained via a convenient four‐step reaction sequence with yields of 66–78 % for the individual steps (Scheme S1). The synthesis of PMCs **1**_***n***_ follows a classical peptide synthesis strategy (Scheme 1). First, the Boc protecting group of **1** is removed under acidic conditions to form the corresponding amine **1‐NH_2_**. Second, the ethyl protecting group of the ester is removed by an aqueous solution of sodium hydroxide to give **1‐COOH**. Last, **1‐NH_2_** and **1‐COOH** are coupled under activation with HBTU/DIPEA to generate dimer **1_2_** in a yield of 45 %. Similarly, by repetitive deprotection and coupling steps with **1‐COOH**, **1_3_** and **1_4_** were obtained in 41 % and 35 % yield, respectively. The fact that the yields remain in the same range despite of chain extension is promising with respect to future research on larger oligomers.

**Scheme 1:**
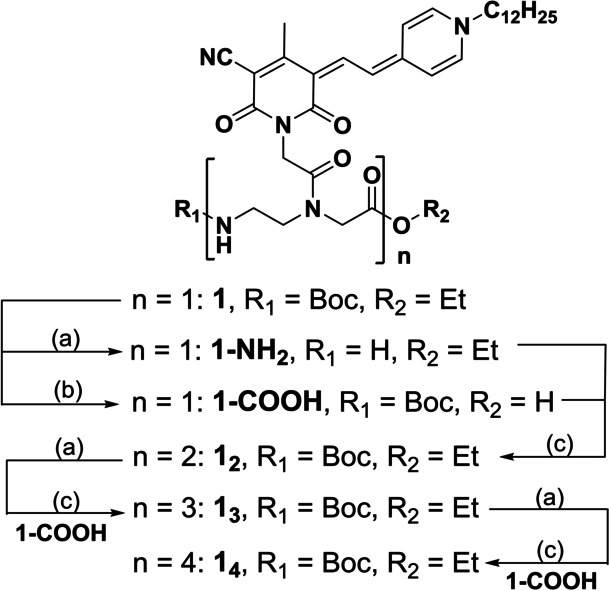
Synthesis of PMC oligomers. Reaction conditions: a) 4 M HCl in dioxane, DCM, 1 h, rt; b) NaOH aq, THF/MeOH, 2 h, rt; c) HBTU, DIPEA, DMF, 3 h, rt.

The self‐assembly properties of **1**, **1_2_**, **1_3_**, and **1_4_** were studied by concentration‐dependent UV/Vis spectroscopy in CHCl_3_ (Figures 2 and S1). Monomer **1** shows only one narrow absorption band at 556 nm at a low concentration of 1.4×10^−7^ M (Figure 2a). With increasing concentration a new hypsochromically shifted band appears at 475 nm that is characteristic for co‐facially stacked MC dimers with H‐type coupled transition dipole moments.[^27^, ^30^] The concomitant decrease of the monomer band and an isosbestic point at 496 nm indicate a two‐state equilibrium process between monomer and dimer species.

**Figure 2:**
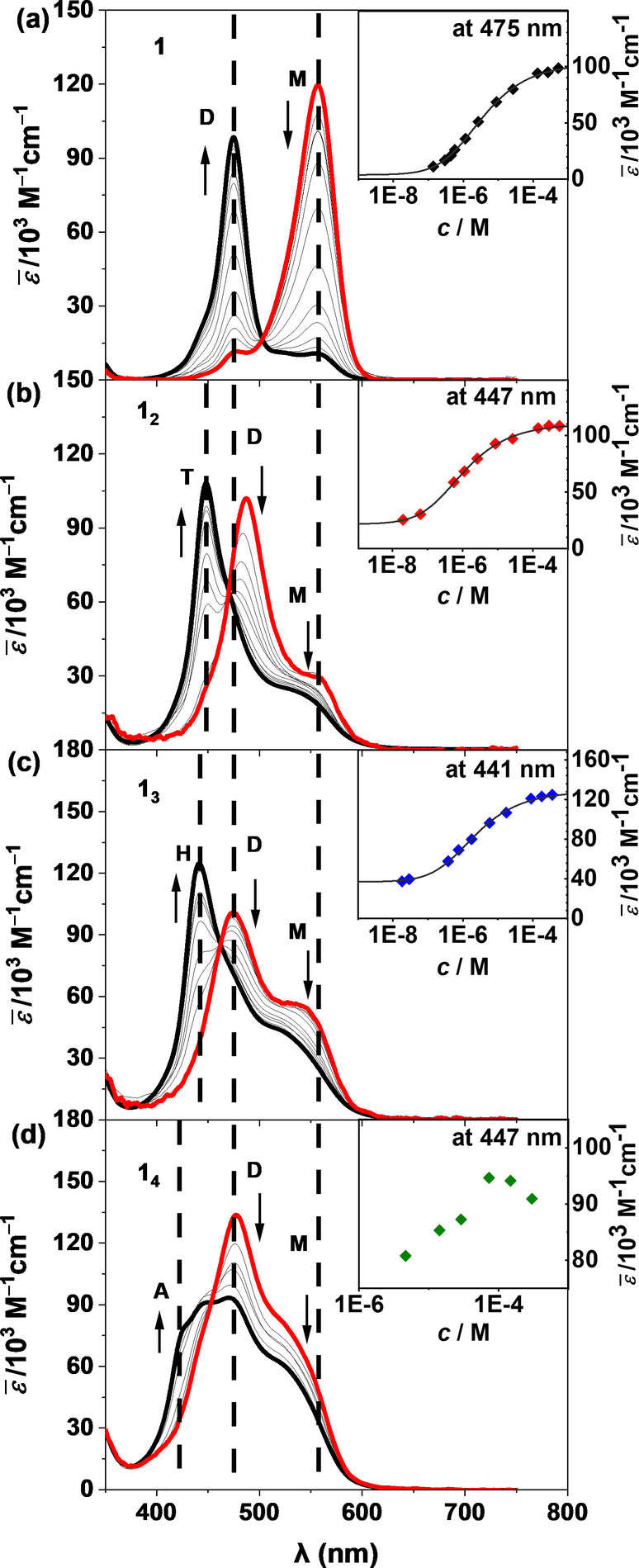
Concentration‐dependent UV/Vis spectra of a) **1** (*c*=1.40×10^−7^–5.29×10^−4^ M), b) **1_2_** (*c*=2.04×10^−8^–5.74×10^−4^ M), c) **1_3_** (*c*=1.81×10^−8^–3.64×10^−4^ M), and d) **1_4_** (*c*=4.65×10^−6^–2.99×10^−4^ M) in CHCl_3_ at 298 K. Black arrows indicate the increase of the aggregate band (dimer band for **1** (D), tetramer band for **1_2_** (T), hexamer band for **1_3_** (H) and undefined aggregate band (A) for **1_4_**) and the decrease in the intensity of the monomer (M) and dimer bands (D) upon increasing concentration. The dashed lines indicate the positions of the absorption maxima of the monomer, dimer and the various aggregate species. The inserts show concentration‐dependent apparent extinction coefficients at 298 K for **1** (at 475 nm), **1_2_** (at 447 nm) and **1_3_** (at 441 nm) at the maximum of the aggregate band in comparison with the simulated curve (solid lines) according to the dimer model. Note: the data for **1_4_** (at 447 nm) could not be properly fitted with dimer model due to the presence of more than two species in the investigated concentration range as indicated by the data points at 447 nm in the inset.

Unlike those simple two absorption bands displayed during the self‐assembly process of monomer **1**, PMC oligomers **1_2_**, **1_3_**, and **1_4_** exhibit already two absorption bands located at 560 nm (monomer‐like) and 480 nm (dimer‐like) even at very low concentrations (Figure 2b–d). The fact that these bands do not change in the lower concentration regime (Figure S2) suggests a partial folding of these oligomers (Figure 3), similar as observed for some previously investigated MC dyes that were interconnected by alkyl chains of variable length.^[31]^ Due to the constraints imparted by the *N*‐(2‐aminoethyl)‐glycine spacer units perfectly antiparallel stack arrangements cannot be achieved for the dipolar MC dyes and accordingly in addition to the most intense hypsochromically shifted band (located close to the dimer band observed for **1**) also J‐type exciton states at lower energy may exhibit notable oscillator strength for such twisted geometries. Furthermore, within the conformational manifold, especially for the longer PMCs **1_3_** and **1_4_**_,_ the presence of partially folded state is most likely that consist of strongly coupled dimeric and weakly coupled monomeric units in equilibrium, leading to the band structure observed in the absorption spectra at low concentrations.^[32]^

**Figure 3:**
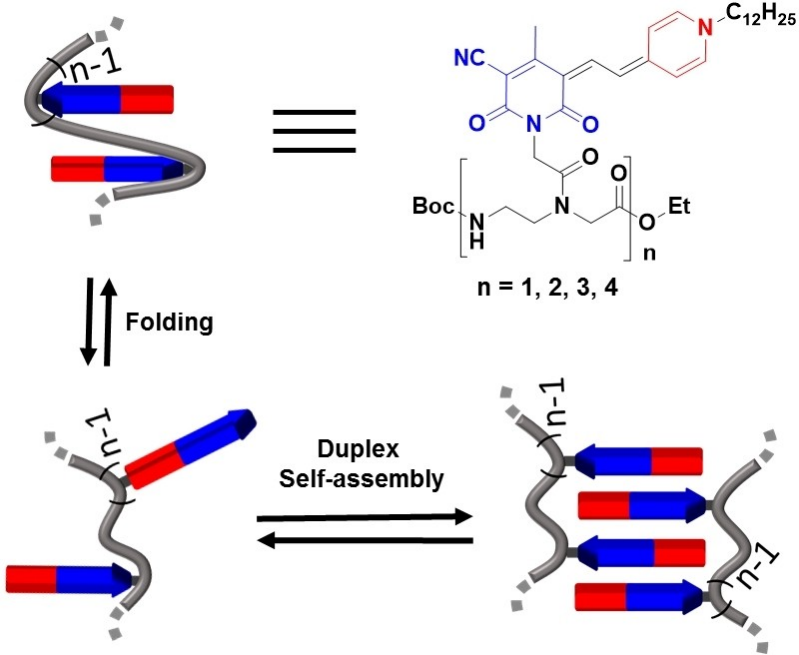
Schematic representation of folding and duplex self‐assembly processes observed for PMC oligomers.^[33]^

As the concentration gradually increases, however, self‐assembly into duplex structures takes place as evidenced by hypsochromically shifted absorption bands at 447 nm and 441 nm for PMCs **1_2_** and **1_3_**, respectively. Thus, as expected for co‐facially stacked dyes,^[34]^ H‐type coupling between neighboring dyes shifts the most intense highest energy exciton state progressively with increasing stack size by 0.38 eV (dimer stack **1**), 0.54 eV (tetramer stack of **1_2_**) and 0.58 eV (hexamer stack of **1_3_**) towards higher energy. For all concentration‐dependent spectra of these oligomers (**1**, **1_2_**, and **1_3_**), we notice a well‐defined isosbestic point in the studied concentration range, indicating a thermodynamic equilibrium between two different species, (folded) monomeric oligomers and duplex aggregates.

The concentration‐dependent UV/Vis spectra of PMCs **1**, **1_2_**, and **1_3_** were fitted with a dimerization model through a global fit algorithm (Figure S1).[^29^, ^35^] The inserts of Figure 2 show the experimental data points of the concentration‐dependent absorbance at the absorption maximum of the duplex (475 nm for **1**, 447 nm for **1_2_**, and 441 nm for **1_3_**) as well as the calculated lines from the global fit analysis. The excellent match of experimental and calculated data throughout the whole concentration range supports our hypothesis that the oligomers self‐assemble in duplex structures. This result is further corroborated by atomic force microscopy (AFM, Figure S10) and DOSY NMR (Figure S12) studies which both indicate the absence of larger aggregate species. For the DOSY experiments the calculated hydrodynamic radii (*r*_H_) of the assemblies (**1**)_2_, (**1_2_**)_2_, and (**1_3_**)_2_ are 10.9, 15.5, and 22.4 Å, respectively. This result further proves that as the length of the oligomer increases, the size of the assembled structures increases. The dimerization constants calculated from our UV/Vis data for the duplex formation of PMCs **1**, **1_2_**, and **1_3_** in CHCl_3_ at 298 K are 3.0×10^5^, 1.1×10^6^, and 3.8×10^5^ M^−1^, respectively. The similar magnitude of these values is at first glance surprising because the number of π–π‐contacts and concomitant dipole‐dipole interactions increases significantly within the series from one (for **1**) to three (**1_2_**) and five (**1_3_**). However, as discussed before, intramolecular dye‐dye contacts originating from folding compete with intermolecular ones as illustrated in Figure 3, thereby reducing the Gibbs energy for duplex formation for the oligomers. Further insights from temperature‐dependent studies in 5 % DMSO/1,4‐dioxane^[36]^ (Figures S3, S4) indeed confirm the enthalpic gain upon duplex formation for the larger oligomers (Table S1) originating from the increased number of perfect antiparallel dye‐dye contacts and the associated entropic cost due to the loss of motional freedom for the dyes and the backbone in the duplex. This leads to steeper melting curves and an increase of the melting temperatures in the series from *T*_m_=296 K for **1** to *T*_m_=340 K for **1_3_** (Figure S4 and Table S1), similarly as observed for the duplex formation between smaller oligonucleotides.^[37]^

The presented results for the smaller oligomers **1**, **1_2_** and **1_3_** revealed supramolecular structures that result from a competition between intramolecular folding and bimolecular self‐assembly that can be controlled either by the concentration or the temperature (Figure 3). At low concentrations or elevated temperature, these oligomers tend to fold intramolecularly while the self‐assembled duplex structure is preferred at higher concentrations and lower temperatures. Because of the large contribution of dipole‐dipole interactions, this competition is also affected by the polarity of the solvent. Thus, for the compared to CHCl_3_ less polar solvent dioxane self‐assembled duplex formation is even more favored whilst upon addition of the polar solvent DMSO the dis‐assembly is observable at constant concentration (Figure S5). For this dissociated state the UV/Vis spectra also clearly indicate the presence of less folded structures with a more pronounced monomer‐like absorption band due to the randomly oriented chromophores. In line with our earlier observations of an aggregation‐induced emission enhancement for this class of dyes upon self‐assembly into a more rigid aggregate state,[^26b^, ^32^, ^35^] solvent‐dependent fluorescence studies (Figure S8 and S9) afforded fluorescence quantum yields of 1.4–1.8 % for the monomeric species of all four compounds in dilute DMSO which increased to 2.6–4.6 % in dilute DMSO/dioxane (1 : 99) where duplex structures (**1**, **1_2_** and **1_3_**) or folded aggregates (**1_4_**, vide infra) prevail.

For **1_4_** we note an obviously different behavior. This was already indicated by the concentration‐dependent UV/Vis spectra shown in Figure 2d and is further corroborated by the solvent‐dependent studies shown in Figure S7. Both data sets suggest a thermodynamically more favored monomer folded state, similar as known for protein folding, which might be due to the multiple options for the formation of antiparallely stacked dye pairs that resists dissociation even in the most dipolar DMSO solvent (Figure S5). Upon decreasing the temperature (Figure S6) or reducing the solvent polarity by addition of methylcyclohexane into CHCl_3_ solutions (Figure S7) the hypsochromically shifted band edge (H‐aggregate) becomes more pronounced, thereby indicating the formation of extended dye stacks by self‐assembly. However, due to a lack of isosbestic points we attribute these changes not to a structurally defined duplex but to polymeric aggregates which is also corroborated by AFM studies (Figure S11). Thus, for **1_4_** the here utilized peptide backbone cannot direct the preferential formation of a MC duplex structure and depending on conditions (temperature, solvent, concentration) various folded and self‐assembled species coexist.

In summary, in this study we reported a new approach toward structurally defined dye aggregates. A series of MC dye oligomers was synthesized by replacing the nucleobases of PNA with dipolar MCs. Driven by the dipole–dipole interactions **1**, **1_2_**, and **1_3_** oligomers self‐assemble into duplex structures containing two, four, and six π‐stacked chromophores under the guidance of the peptide backbone. The competing processes of intramolecular folding and intermolecular self‐assembly for these oligomers can be controlled through appropriate conditions (solvent, temperature and concentration). We envision that duplex structures can also be accomplished for other dyes connected to the *N*‐(2‐aminoethyl)‐glycine‐based pseudopeptide backbones and that subtle modifications of this backbone may also enable the duplex formation for highly desired extended oligomer strands bearing eight, ten or more π‐stacked chromophores. However, already the herein reported tetra‐ and hexamer stacks should be quite useful, for instance by incorporating them within DNA backbones where their highly structure‐dependent exciton coupling can sense structural changes upon hybridization,^[25b]^ hairpin formation or catalysis.^[38]^

## Conflict of interest

The authors declare no conflict of interest.

## Supporting information

As a service to our authors and readers, this journal provides supporting information supplied by the authors. Such materials are peer reviewed and may be re‐organized for online delivery, but are not copy‐edited or typeset. Technical support issues arising from supporting information (other than missing files) should be addressed to the authors.

Supporting Information

## Data Availability

The data that support the findings of this study are available from the corresponding author upon reasonable request.
