# Supplementary material for: Peptide Backbone Directed Self‐Assembly of Merocyanine Oligomers into Duplex Structures
Source: Angew Chem Int Ed Engl. 2022 Mar 23;61(21):e202200120. doi: 10.1002/anie.202200120 (PMC9401582; doi:10.1002/anie.202200120)
Supplement: Supplementary file 1 — Supporting Information [file ANIE-61-0-s001.pdf]

Angewandte Chemie

Eine Zeitschrift der Gesellschaft Deutscher Chemiker

Supporting Information

Peptide Backbone Directed Self-Assembly of Merocyanine Oligomers into Duplex Structures

B. Liu, Y. Vonhausen, A. Schulz, C. Höbartner, F. Würthner**

Supporting Information

Table of contents

1. Materials and methods 2

2. Synthesis and characterization 3

3. UV/Vis and fluorescence spectroscopy 9

4. AFM analysis 17

5. DOSY NMR analysis 18

6. NMR spectra 19

7. MS spectra 25

References 29

1. Materials and methods

All solvents and reagents were purchased from commercial sources and used as received without further purification. Column chromatography was performed using silica gel 60 M (0.04-0.063 mm). Size exclusion column chromatography was performed with commercial glass columns using Bio-Beads SX1 with 10% methanol/DCM as an eluent. NMR spectra were recorded on a Bruker Advance III HD 400 or Bruker Advance III HD 600 spectrometers at 295 K. The spectra were calibrated to the residual solvent peak and the chemical shifts δ are in ppm. Multiplicities are denoted as follows: s = singlet, d = doublet, t = triplet, dd = doublet of doublet, td = triplet of doublet, m = multiplet, br = broad. High-resolution mass spectra (ESI) were recorded on an ESI MicrOTOF Focus spectrometer. Solvents for spectroscopic studies were of spectroscopic grade and used as received. UV/Vis spectra were measured on a JASCO V-670 or V-770 spectrometer at 298 K in conventional quartz cell cuvettes with path lengths of 1–100 mm. For concentration-dependent studies, the samples were prepared one day before measurement. Emission and excitation spectra were measured with a FLS980-D2D2-ST (Edinburgh Instruments Ltd., UK) fluorescence spectrometer and corrected against the photomultiplier sensitivity and the lamp intensity. The fluorescence quantum yields (Φ_f) were determined relative as average value of four different excitation wavelengths under highly diluted (OD ≤ 0.05) and magic angle conditions (54.7°). Melting points were determined with a BÜCHI Melting Point B-545 apparatus and are uncorrected. Note: the restricted rotation around the amide bond connecting the substitution and the acetamide backbone results in the coexistence of two stable conformers as also observed in the case of PNAs.[1,2]

2. Synthesis and characterization

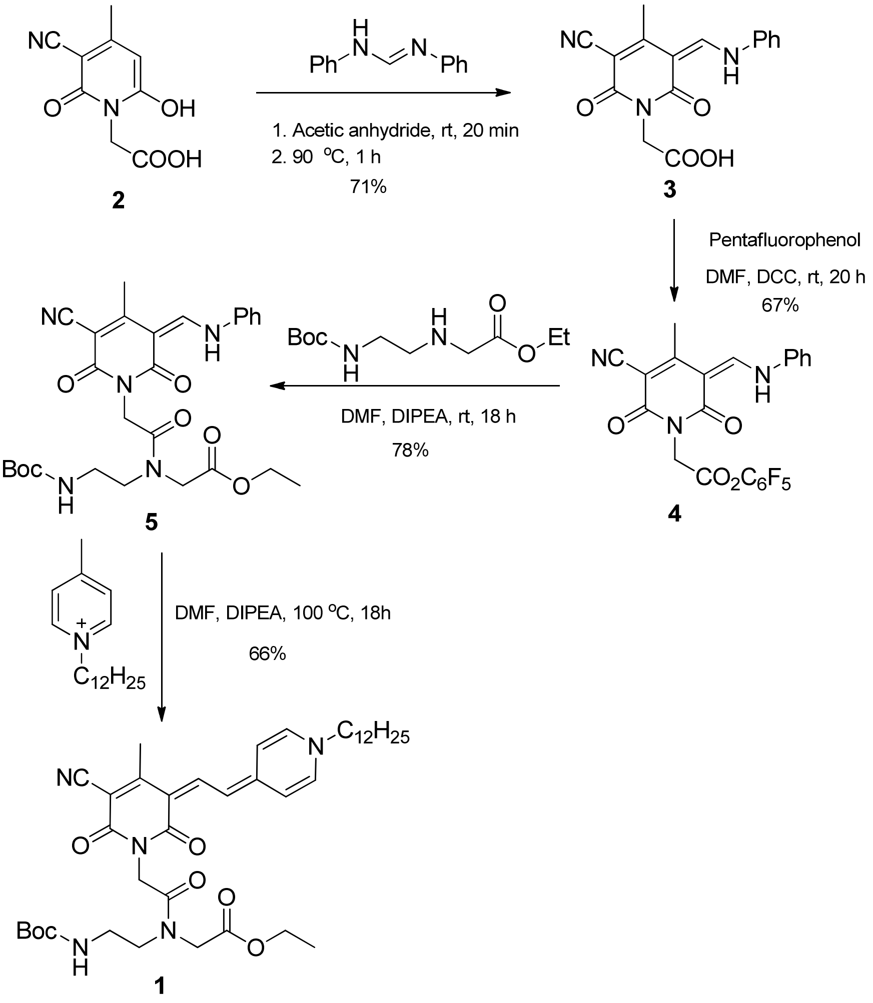

Scheme S1. Synthesis of merocyanine monomer **1**.

Compound **2**,[3] 1-dodecyl-4-methylpyridin-1-ium[4] and ethyl 2-((2-((*tert*-butoxycarbonyl)amino)ethyl)amino)acetate[5] were synthesized according to the reported procedures.

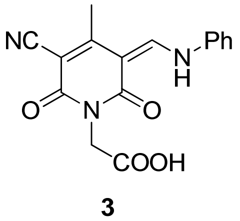

3

Compound **2** (1.04 g, 5 mmol) and *N*,*N*'-diphenylformamidine (0.98 g, 5 mmol) were suspended in 15 mL acetic anhydride. The suspension was stirred at room temperature for 20 min and then at 90 °C for 1 h to complete the reaction. The reaction mixture was then cooled to room temperature and 50 mL diethyl ether were added. The resulting solid was collected by filtration and washed with 50 mL diethyl ether. After drying in vacuum, a yellowish powder was obtained

(1.10 g, 71%). Mp.: 287 - 288 °C. ^{1}H NMR (400 MHz, DMSO-d_6): δ (ppm) = 13.04 (s, 1H), 12.79 (d, J = 13.8 Hz, 1H), 8.65 (d, J = 13.8 Hz, 1H), 7.78 – 7.68 (m, 2H), 7.56 – 7.48 (m, 2H), 7.36 (t, J = 8.4 Hz, 1H), 4.56 (s, 2H), 2.62 (s, 3H). ^{13}C NMR (101 MHz, DMSO-d_6): δ (ppm) = 169.7, 164.7, 161.3, 161.2, 153.9, 138.5, 130.1, 127.5, 120.2, 116.7, 100.0, 93. 8, 40.9, 18.0. HRMS (ESI): m/z calcd for $C_{16}H_{13}N_3NaO_4$ [M + Na]$^+$: 334.0798, found: 334.0804.

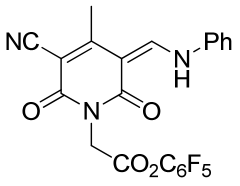

4

Compound **3** (0.62 g, 2 mmol) and pentafluorophenol (0.55 g, 3 mmol) were dissolved in 20 mL DMF and the solution was stirred at 0 °C for 15 min. DCC (0.62 g, 3 mmol) was added, the resulting mixture was stirred at 0 °C for 15 min and at room temperature overnight. The solvent was removed under vacuum and the crude product was suspended in 5 mL cold methanol. The product was collected by filtration, washed with 10 mL cold methanol, and obtained as a light yellow powder (0.64 g, 67%). Mp.: 225 - 226 °C. ^{1}H NMR (400 MHz, DMSO-d_6): δ (ppm) = 12.80 (d, J = 13.4 Hz, 1H), 8.66 (d, J = 12.8 Hz, 1H), 7.70 (d, J = 8.0, 2H), 7.50 (t, J = 8.4, 2H), 7.34 (t, J = 7.4 Hz, 1H), 4.56 (s, 2H), 2.62 (s, 3H). ^{13}C NMR (101 MHz, DMSO-d_6): δ (ppm) = 165.4, 164.3, 162.1, 161.0, 154.6, 138.5, 130.1, 127.7, 120.4, 120.2, 116.5, 99.8, 93.4, 40.4, 18.19. HRMS (ESI): m/z calcd for $C_{22}H_{12}F_5N_3NaO_4$ [M + Na]$^+$: 500.0640, found: 500.0627.

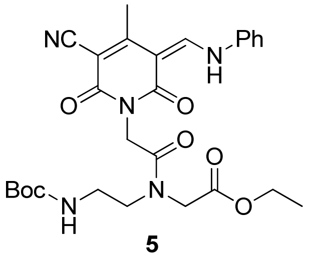

5

Compound **4** (7.15 g, 15 mmol), 2-((2-((*tert*-butoxycarbonyl)amino) ethyl)amino)acetate (3.69 g, 15 mmol) and DIPEA (1.94 g, 15 mmol) were dissolved in 120 mL DMF and the solution was stirred at room temperature overnight. The solvent was removed under vacuum and the crude product was purified by column chromatography (eluent: DCM/MeOH = 98/2, v/v%), followed by removal of solvent to afford **5** as a light yellow powder (6.30 g, 78%). Mp.: 106 - 107 °C. ^{1}H NMR (400 MHz, DMSO-d_6): δ (ppm) = 12.83 (d, J = 13.5 Hz, 1H, conformer a), 12.81 (d, J = 13.5 Hz, 1H, conformer b), 8.65 (d, J = 13.5 Hz, 1H, conformer a), 8.63 (d, J = 13.5 Hz, 1H, conformer b), 7.66-7.70 (m, 4H), 7.51 (t, J = 7.5 Hz, 4H), 7.33 (t, J = 7.5 Hz, 2H), 6.94 (t, J = 7.5 Hz, 1H, conformer a), 6.76 (t, J = 7.5 Hz, 1H, conformer b), 4.82 (s, 2H, conformer a), 4.69 (s, 2H, conformer b), 4.41 (s, 2H, conformer b), 4.21 (q, J = 7.1 Hz, 2H, conformer b), 4.08 (q, J = 7.1

Hz, 2H, conformer a), 4.04 (s, 2H, conformer a), 3.51 (t, *J* = 6.7 Hz, 2H, conformer a), 3.32 (t, *J* = 7.0 Hz, 2H, conformer b), 3.21 (dd, *J* = 12.6, 6.4 Hz, 2H, conformer a), 3.01 (dd, *J* = 12.7, 6.3 Hz, 2H, conformer b), 2.62 (s, 6H), 1.40 (s, 9H, conformer a), 1.37 (s, 9H, conformer b), 1.28 (t, *J* = 7.5 Hz, 3H, conformer b), 1.18 (t, *J* = 7.5 Hz, 3H, conformer a). ^{13}C NMR (101 MHz, DMSO-d_6): δ (ppm) = 169.9, 169.6, 167.4, 167.1, 164.9, 164.8, 161.2, 161.1, 161.1, 161.0, 156.2, 153.7, 138.5, 138.5, 130.1, 130.1, 127.4, 120.1, 120.0, 116.8, 100.1, 100.0, 94.0, 78.5, 78.2, 61.6, 61.0, 49.6, 48.2, 47.5, 40.5, 38.8, 28.6, 18.0, 14.5. HRMS (ESI): m/z calcd for $C_{27}H_{33}N_5NaO_7$ [M + Na]$^+$: 562.2272, found: 562.2272.

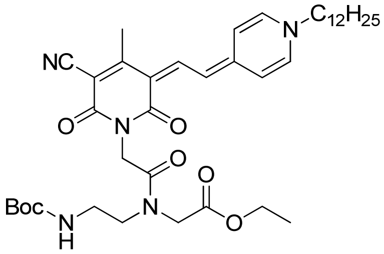

1

Compound **5** (2.70 g, 5 mmol), 1-dodecyl-4-methylpyridin-1-ium (1.70 g, 5 mmol) and DIPEA (0.65 g, 5 mmol) were dissolved in 120 mL DMF and the solution was stirred at 100 °C overnight. The solvent was removed under vacuum and the crude product was purified by column chromatography (eluent: DCM/MeOH = 97/3, v/v%), followed by removal of the solvent to afford **1** as a dark red powder (2.3 g, 66%). Mp.: 136 - 137 °C. ^{1}H NMR (400 MHz, DMSO-d_6): δ (ppm) = 8.47 (d, *J* = 7.1 Hz, 4H), 7.83 (d, *J* = 5.9 Hz, 4H), 7.74 (s, 4H), 6.92 (t, *J* = 5.7 Hz, 1H, conformer a), 6.74 (t, *J* = 5.7 Hz, 1H, conformer b), 4.74 (s, 2H, conformer a), 4.59 (s, 2H, conformer b), 4.37 (s, 2H, conformer b), 4.28 (t, *J* = 7.2 Hz, 4H), 4.20 (q, *J* = 7.1 Hz, 2H, conformer b), 4.08 (q, *J* = 7.1 Hz, 2H, conformer a), 4.02 (s, 2H, conformer a), 3.49 (t, *J* = 6.9 Hz, 2H, conformer a), 3.30 (t, *J* = 7.0 Hz, 2H, conformer b), 3.21 (dd, J_1 = 13.0, J_2 = 6.4 Hz, 2H, conformer a), 3.01 (dd, J_1 = 13.1, J_2 = 6.5 Hz, 2H, conformer b), 2.44 (s, 6H), 1.81-1.84 (m, 4H), 1.39 (s, 9H, conformer a), 1.37 (s, 9H, conformer b), 1.17-1.28 (m, 42H), 0.83-0.85 (m, 6H). ^{13}C NMR (101 MHz, DMSO-d_6): δ (ppm) = 170.1, 169.8, 168.6, 168.4, 162.7, 162.5, 156.4, 156.2, 155.9, 142.7, 138.8, 121.2, 120.1, 114.9, 105.1, 85.1, 78.4, 78.2, 61.5, 60.9, 58.7, 55.4, 49.7, 48.2, 47.6, 38.9, 31.8, 30.9, 29.5, 29.4, 29.3, 29.2, 28.9, 28.6, 25.9, 22.6, 18.9, 14.5, 14.4. HRMS (ESI): m/z calcd for $C_{39}H_{57}N_5NaO_7$ [M + Na]$^+$: 730.4150, found: 730.4163.

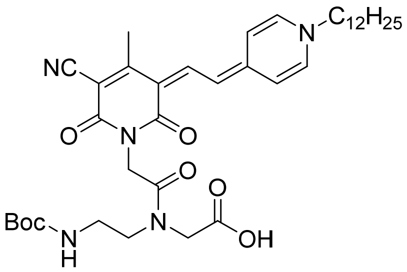

1-COOH

Monomer **1** (0.7 g, 1 mmol) was dissolved in a solution of 10 mL methanol and 10 mL THF, and 2 mL 4 M NaOH aqueous solution was slowly added into the solution at room temperature. The resulting solution was stirred at room temperature for 2 hours until TLC showed no starting materials. After that, 50 mL DCM was added and the organic layer was separated and washed with water (3 x 30 mL). The organic layer was then dried with Na_2SO_4 and the solvents were removed under vacuum to yield **1-COOH** as a dark red solid (the product was used for the next step without further purification).

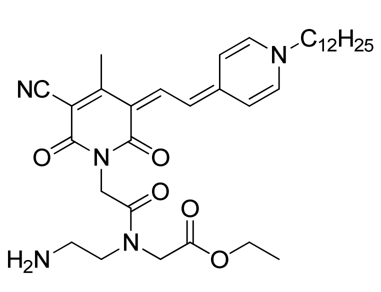

1-NH$_2$

Monomer **1** (0.7 g, 1 mmol) was dissolved in 30 mL DCM and 4 mL HCl (4 M in 1,4-dioxane) was added slowly. The solution was stirred at room temperature for 1 hour. The solvent was removed under vacuum and the crude product was suspended in 20 mL diethyl ether. The product was collected by filtration to afford **1-NH$_2$** as a dark red powder (the product was used for the next step without further purification).

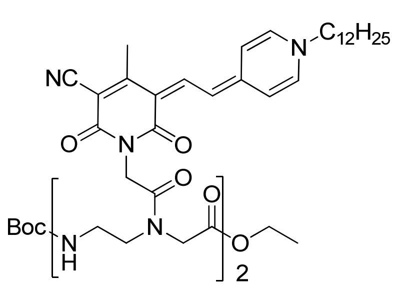

1$_2$

Compounds **1-NH$_2$** (0.20 g, 0.3 mmol), **1-COOH** (0.20 g, 0.29 mmol) and HBTU (0.23 g, 0.6 mmol) were dissolved in 40 mL DMF, followed by addition of DIPEA (0.12 g, 0.93 mmol). The solution was stirred at room temperature for 3 hours. The solvent was removed under vacuum and the crude product was purified by size exclusion column chromatography three times, followed by removal of the solvent to afford **1$_2$** as a dark red powder (0.18 g, 45%). Mp.: 168 - 169 °C. ^{1}H NMR (400 MHz, DMSO-*d$_6$*): δ (ppm) = 8.46-8.48 (m, 4H), 7.79-7.83 (m 4H), 7.68-7.73 (m, 4H), 6.92-6.88 (m, 1H), 4.59-4.74 (m, 4H), 4.26-4.29 (m, 4H), 3.99-4.07 (m, 4H), 3.62-3.58 (m, 1H), 3.46-3.47 (m, 2H), 3.30-3.32 (m, 1H), 3.15-3.18 (m, 2H), 3.01-3.05 (m, 1H), 2.39-

2.51 (m, 6H), 1.81-1.83 (m, 4H), 1.35-1.39 (m, 9H), 1.15-1.25 (m, 43H), 0.83-0.86 (m, 6H). HRMS (ESI): m/z calcd for $C_{71}H_{100}N_{10}NaO_{11}$ $[M + Na]^+$: 1291.7465, found: 1291.7441.

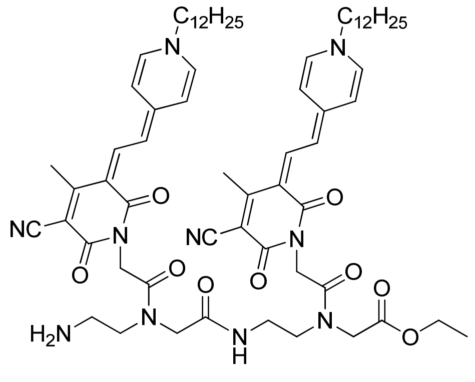

1₂-NH₂

Compound **1₂** (0.1 g, 0.08 mmol) was dissolved in 20 mL DCM and 2 mL HCl (4 M in 1,4-dioxane) was added slowly. The solution was stirred at room temperature for 1 hour. The solvent was removed under vacuum to afford **1₂-NH₂** as a dark red solid (the product was used for the next reaction without further purification).

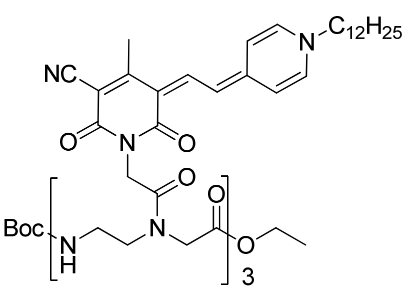

1₃

Compounds **1₂-NH₂** (100 mg, 0.07 mmol), **1-COOH** (100 mg, 0.15 mmol) and HBTU (115 mg, 0.3 mmol) were dissolved in 40 mL DMF, followed by addition of DIPEA (60 mg, 0.46 mmol). The solution was stirred at room temperature for 3 hours. The solvent was removed under vacuum and the crude product was purified by size exclusion column chromatography three times, followed by removal of the solvent to afford **1₃** as a dark red powder (60 mg, 41%). Mp.: 196 - 197 °C. ^{1}H NMR (400 MHz, DMSO-d_6): δ (ppm) = 8.45-8.47 (m, 6H), 7.64-7.82 (m, 12H), 6.87-6.82 (m, 1H), 4.73-4.53 (m, 6H), 4.50-4.41 (m, 2H), 4.23-4.28 (m, 7H), 4.01-4.09 (m, 5H), 3.55-3.62 (m, 6H), 3.11-3.13 (m, 1H), 2.99-3.01 (m, 1H), 2.37-2.44 (m, 9H), 1.80-1.83 (m, 6H), 1.33-1.37 (m, 9H), 1.15-1.23 (m, 63H), 0.83-0.86 (m, 9H). HRMS (ESI): m/z calcd for $C_{103}H_{143}N_{15}NaO_{15}$ $[M + Na]^+$: 1853.0780, found: 1853.0733.

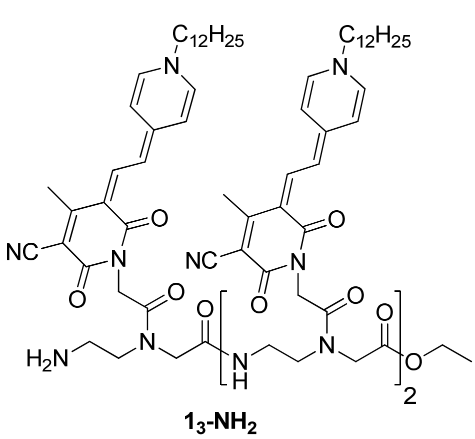

1₃-NH₂

Compound **1₃** (90 mg, 0.05 mmol) was dissolved in 20 mL DCM and 2 mL HCl (4 M in 1,4-dioxane) was added slowly. The solution was stirred at room temperature for 2 hours. The solvent was then

removed under vacuum to afford **1_3-NH$_2$** as a dark red solid (the product was used for the next step without further purification).

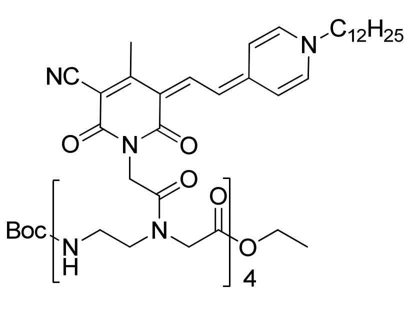

1_4

Compounds **1_3-NH$_2$** (50 mg, 0.03 mmol), **1-COOH** (50 mg, 0.07 mmol) and HBTU (75 mg, 0.15 mmol) were dissolved in 20 mL DMF, followed by addition of DIPEA (30 mg, 0.23 mmol). The solution was stirred at room temperature for 3 hours. The solvent was removed under vacuum and the crude product was purified by size exclusion column chromatography five times, followed by removal of the solvent to afford **1_4** as a dark red powder (23 mg, 35%). Mp.: 214 - 215 °C. ^{1}H NMR (400 MHz, DMSO-d_6): δ (ppm) = 8.43-8.46 (m, 8H), 7.70-7.78 (m, 16H), 6.85-6.81 (m, 1H), 4.44-4.73 (m, 10H), 4.24-4.27 (m, 9H), 4.02-4.07 (m, 6H), 3.80-3.95 (m, 2H), 3.49-3.60 (m, 6H), 2.89-3.15 (m, 4H), 2.33-2.41 (m, 12H), 1.78-1.83 (m, 8H), 1.33-1.36 (m, 9H), 1.11-1.24 (m, 79H), 0.83-0.88 (m, 16H). HRMS (ESI): m/z calcd for $C_{135}H_{186}N_{20}Na_2O_{19}$ [M + 2Na]$^{2+}$: 1218.6994, found: 1218.6977.

Global fit analysis for duplex formation

A global fit algorithm as described in detail in our earlier work[6-8] was applied for the evaluation of the concentration-dependent UV/Vis data according to the dimer model.

The equilibrium between monomer (M) and dimer (D) is given by M + M ⇌ D. The dimerization constant K_2 can be calculated by

$$K_2 = \frac{c_D}{{c_M}^2} \qquad \text{(S1)}$$

where c_M and c_D are the monomer and dimer concentration, respectively.

The molar concentration is given by the sum of the concentration of the monomers and dimers:

$$c = c_M + 2c_D \qquad \text{(S2)}$$

3. UV/Vis and fluorescence spectroscopy

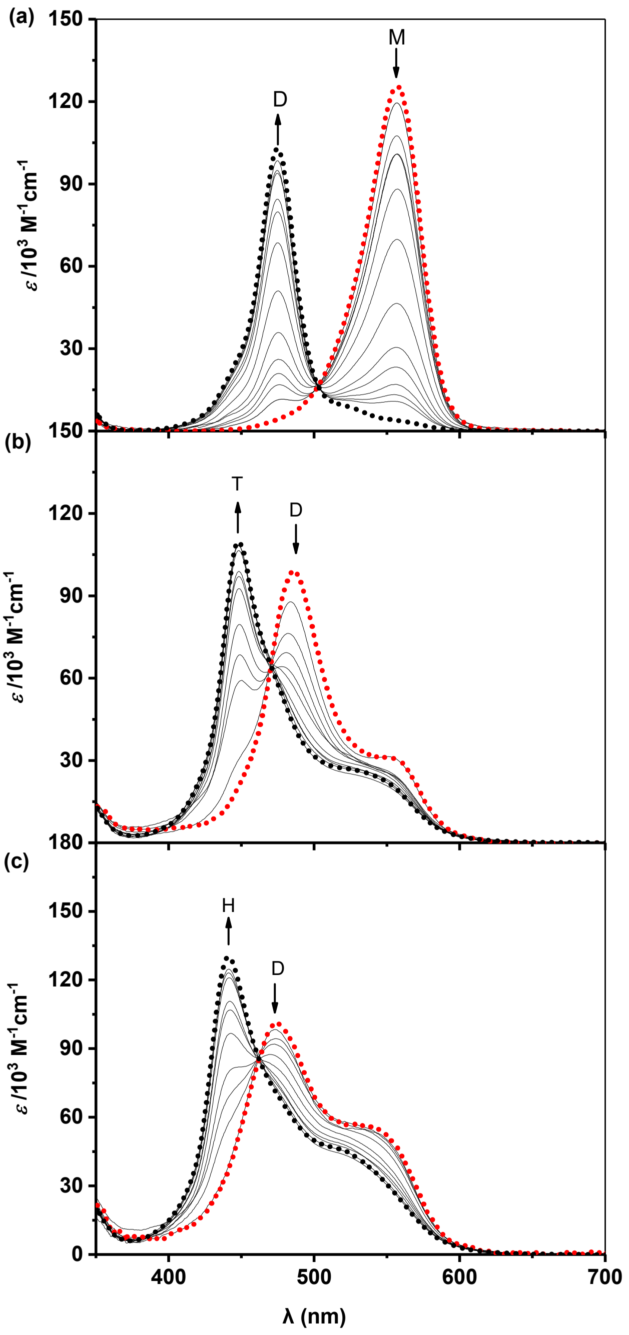

Fig. S1. Concentration-dependent UV/vis spectra of (a) **1** (c = 1.40 × 10^{-7} – 5.29 × 10^{-4} M), (b) $\mathbf{1_2}$ (c = 2.04 × 10^{-8} – 5.74 × 10^{-4} M) and (c) $\mathbf{1_3}$ (c = 1.81 × 10^{-8} – 3.64 × 10^{-4} M) in $CHCl_3$ at 298 K. Black arrows indicate the increase of the aggregate band (dimer band for **1**, tetramer band for $\mathbf{1_2}$ and hexamer band for $\mathbf{1_3}$) and the decrease in the intensity of the monomer and dimer bands upon decreasing concentration. Dotted lines represent the spectra of the individual species obtained by global fit analysis for the (folded) monomers (red) and the duplexes (black) according to the dimerization model.

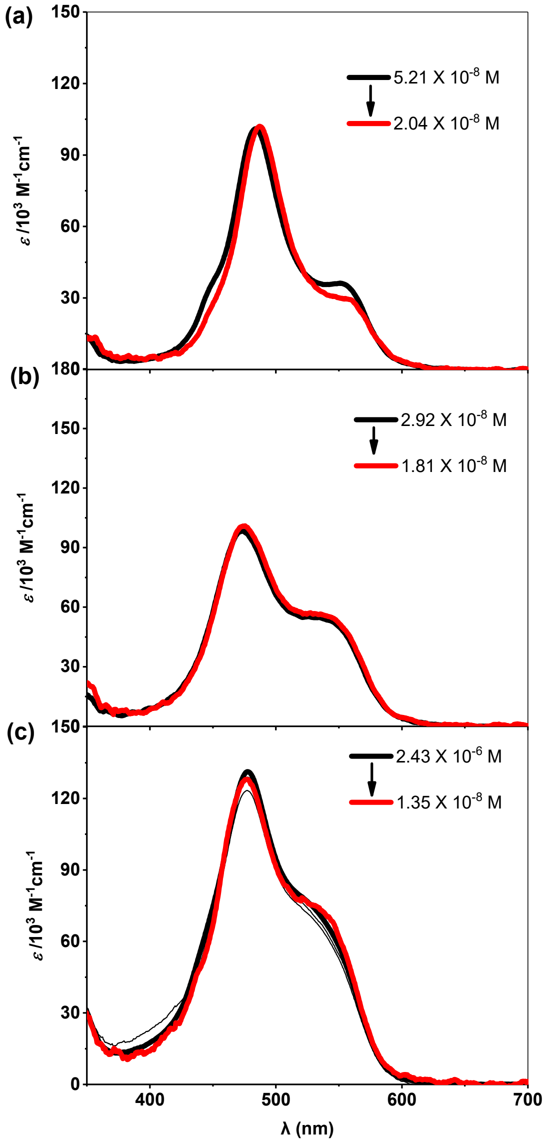

Fig. S2. Concentration-dependent UV/vis spectra of $\mathbf{1_2}$ (c = 5.21 × 10^{-8} – 2.04 × 10^{-8} M) $\mathbf{1_3}$ (c = 2.92 × 10^{-8} – 1.81 × 10^{-8} M), and $\mathbf{1_4}$ (c = 2.43 × 10^{-6} – 1.35 × 10^{-8} M) in $CHCl_3$ at 298 K.

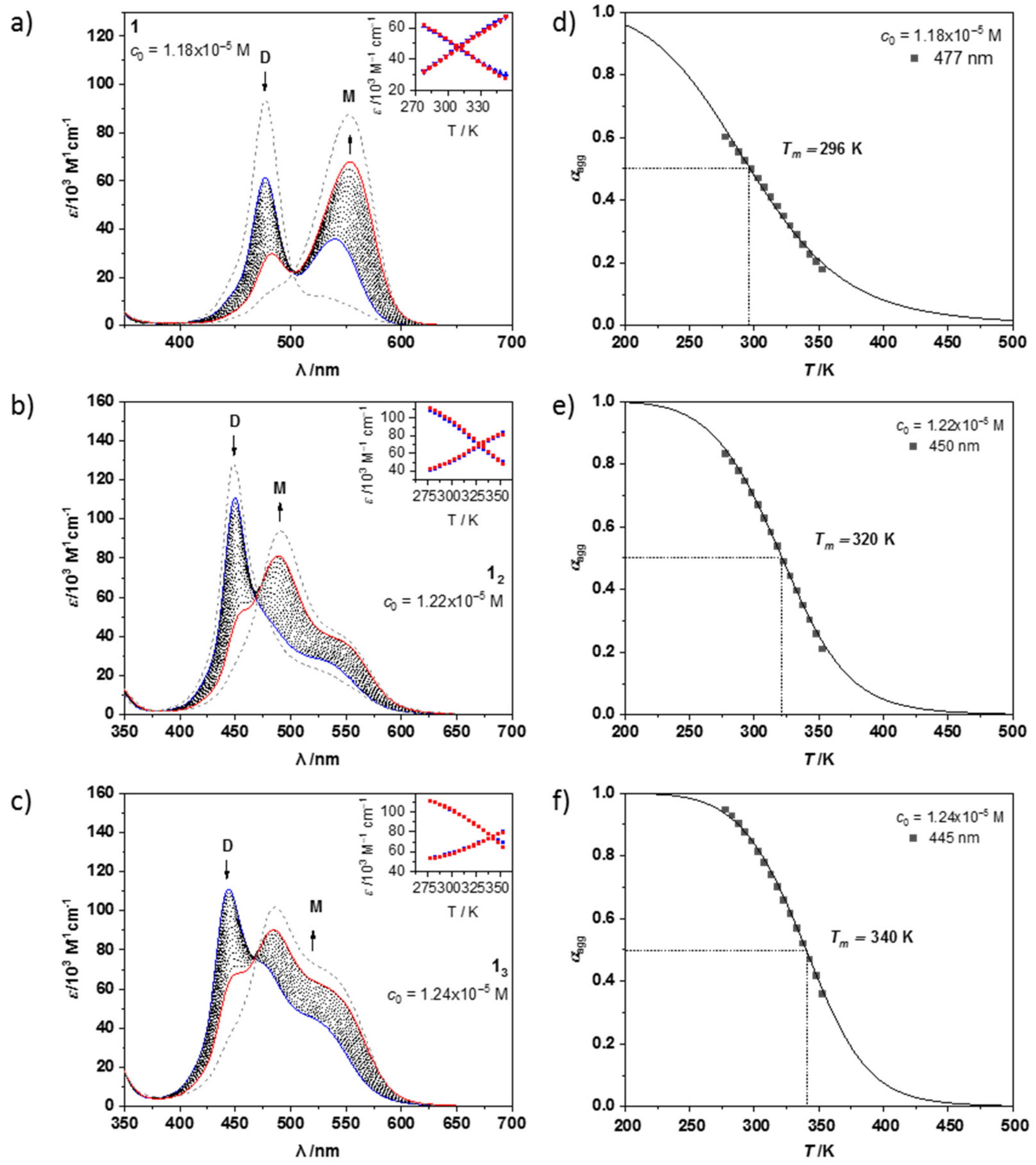

Fig. S3. a-c) Temperature-dependent UV/Vis spectra (2.5 K /min) of a) **1** (c_0 = 1.18 x 10^{-5} M), b) $\mathbf{1_2}$ (c_0 = 1.22 x 10^{-5} M) and c) $\mathbf{1_3}$ (c_0 = 1.24 x 10^{-5} M) in 5% DMSO/1,4-dioxane from 278 K (blue line) to 353 K (red line). The spectra representing the pure duplex (D) and monomer (M) states are shown as well with grey dashed lines as measured for (a) c_0 = 1.15 x 10^{-3} M at 278 K (D) and c_0 = 1.20 x 10^{-6} M at 353 K (M); b) c_0 = 1.18 x 10^{-3} M at 278 K (D) and c_0 = 1.24 x 10^{-6} M at 353 K (M); c) c_0 = 1.25 x 10^{-6} M at 353 K (M)). Insets show the temperature-dependent apparent molar extinction at two selected wavelengths for the cooling (blue symbols) and heating (red symbols) cycle, confirming the fast equilibration (no hysteresis). d-f) Temperature-dependent degree of aggregation calculated at the wavelength of the maximum of the D absorption band. Since for $\mathbf{1_3}$ no experimental spectrum of the fully aggregated state could be obtained due to precipitation at higher concentrations $\varepsilon(\mathrm{D})_{445\mathrm{nm}}$ ~ 115000 M^{-1}cm^{-1} was estimated for the calculation of α_{agg}. Data were fitted according to the temperature-dependent dimer model (eq. (S4)).

Equation (S3) was used to calculate the temperature-dependent degree of aggregation from the experimental UV/Vis spectra. The data were fitted according to the dimer self-assembly model[9] by expressing the temp.-dependence of the binding constant K by $K = \exp(-\Delta G/(RT))$ and $\Delta G = \Delta H - T{*}\Delta S$. With ΔG as the Gibbs free energy gain upon duplex formation and ΔH and ΔS as the temperature-independent binding enthalpy and entropy, respectively. The melting temperatures were extracted at $\alpha_{\mathrm{agg}} = 0.5$.

$$\alpha_{\mathrm{agg}} = \frac{\varepsilon(\lambda)-\varepsilon_{\mathrm{M}}(\lambda)}{\varepsilon_{\mathrm{D}}(\lambda)-\varepsilon_{\mathrm{M}}(\lambda)} \qquad \text{(S3)}$$

$$\alpha_{\mathrm{agg}} = \frac{4Kc_0+1-\sqrt{8Kc_0+1}}{4Kc_0} = \frac{4\ e^{\frac{-(\Delta H-T*\Delta S)}{RT}}c_0+1-\sqrt{8\ e^{\frac{-(\Delta H-T*\Delta S)}{RT}}c_0+1}}{4\ e^{\frac{-(\Delta H-T*\Delta S)}{RT}}c_0} \qquad \text{(S4)}$$

Table S1. Melting temperatures (Tm) and thermodynamic parameters calculated for the duplex formation from the data shown in Figure S3 and analysis according to equation (S4).

	**1 at** **c = 1.18 x 10^{-5} M** **477 nm**	**1_2 at** **c = 1.22 x 10^{-5} M** **450 nm**	**1_3 at** **c = 1.25 x 10^{-5} M** **445 nm**
T_{m} /K	296	320	340
ΔH /kJ mol^{-1}	–29.0±0.7	–48.9±0.5	–58.8±0.6
ΔS /J mol^{-1} K^{-1}	–2.9±2	–58±1	–79±2
ΔG (293 K) /kJ mol^{-1}	–28.2	–31.9	–35.6
K (293 K) /M^{-1} cm^{-1}	1.1 x 10^5	4.9 x 10^5	2.2 x 10^6

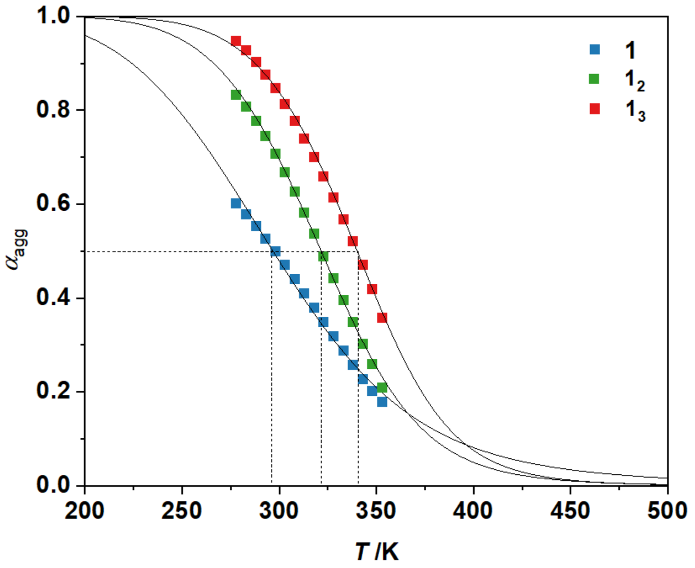

Fig. S4. Comparison of the temperature-dependent degree of aggregation of **1**, **1_2** and **1_3** (c_0 = 1.2 x 10^{-5} M, 5%DMSO/1,4-dioxane).

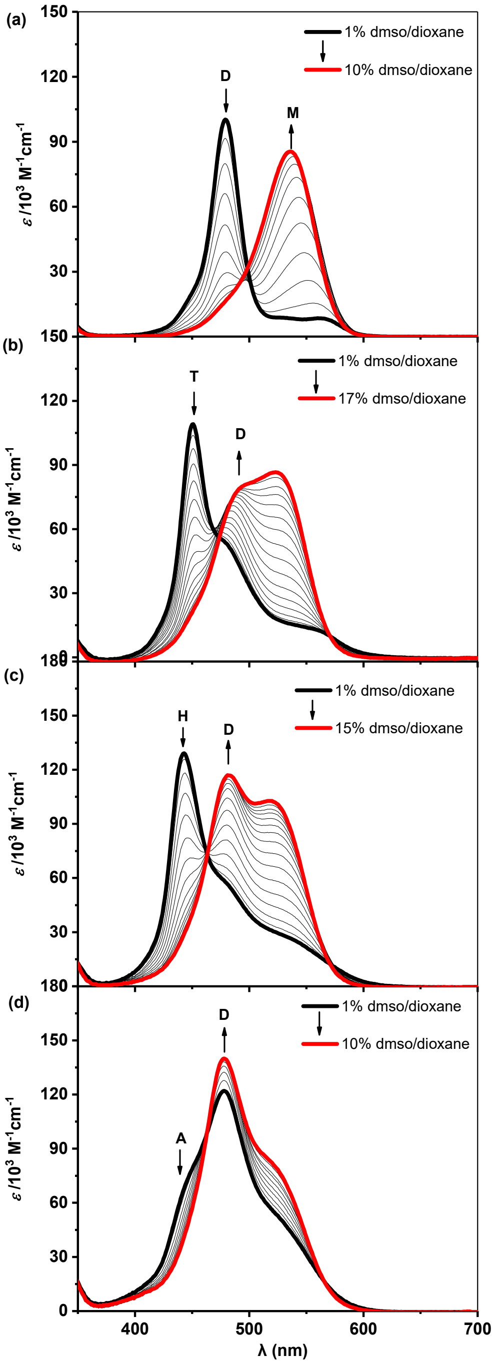

Fig. S5. UV-vis spectra for the dis-assembly of the self-assembled oligomers **1-1$_4$**. PMC oligomers **1-1$_4$** were dissolved in DMSO at 293 K at a concentration of (a) [**1**] = 4.33 x 10^{-4} M, (b) [**1$_2$**] = 2.33 x 10^{-4} M, (c) [**1$_3$**] = 1.96 x 10^{-4} M, (d) [**1$_4$**] = 3.94 x 10^{-4} M and diluted 100 times in nonpolar dioxane to afford the self-assembled duplexes (black bold line). The dis-assembly processes were accomplished by stepwise addition of the dipolar solvent DMSO into these solutions (thin black lines) to afford the fully dis-assembled state (bold red line). Arrows indicate the decrease in the intensity of the aggregate band and the appearance of the characteristic bands for intramolecularly folded dimer and unfolded monomer units upon addition of DMSO.

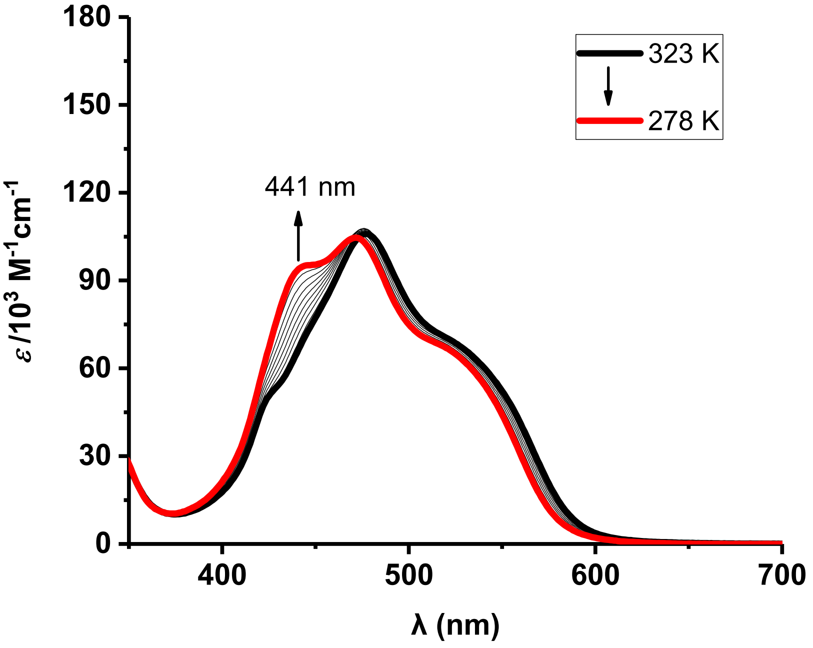

Fig. S6. Temperature-dependent UV/Vis spectra of $\mathbf{1_4}$ ($CHCl_3$, 3.81×10^{-5} M) from 323 to 278 K.

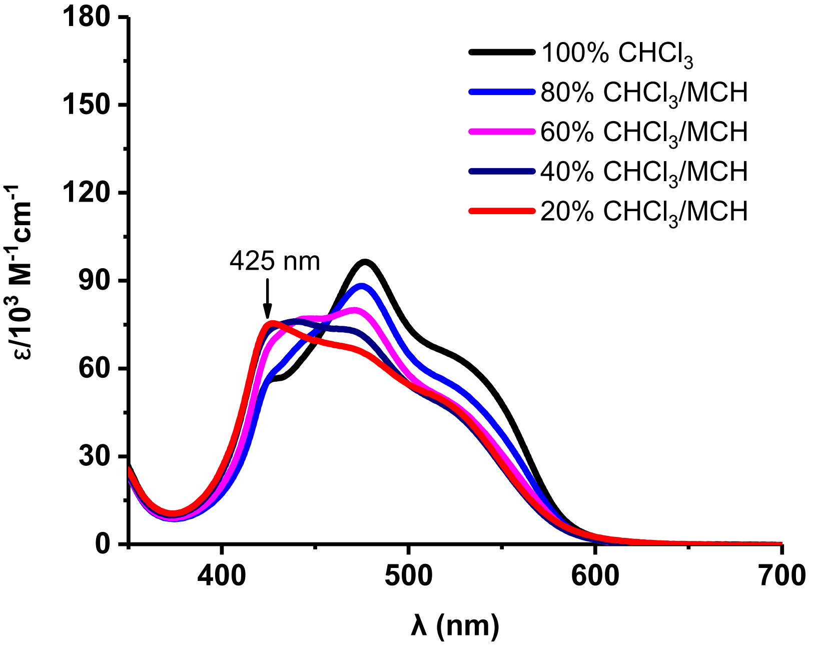

Fig. S7. Solvent-dependent UV/Vis spectra of [$\mathbf{1_4}$] = 3.41 x 10^{-6} M in $CHCl_3$/MCH mixtures (T=298 K) starting from pure $CHCl_3$ (black lines) and successively increasing the volume fraction of MCH up to 80% (red lines).

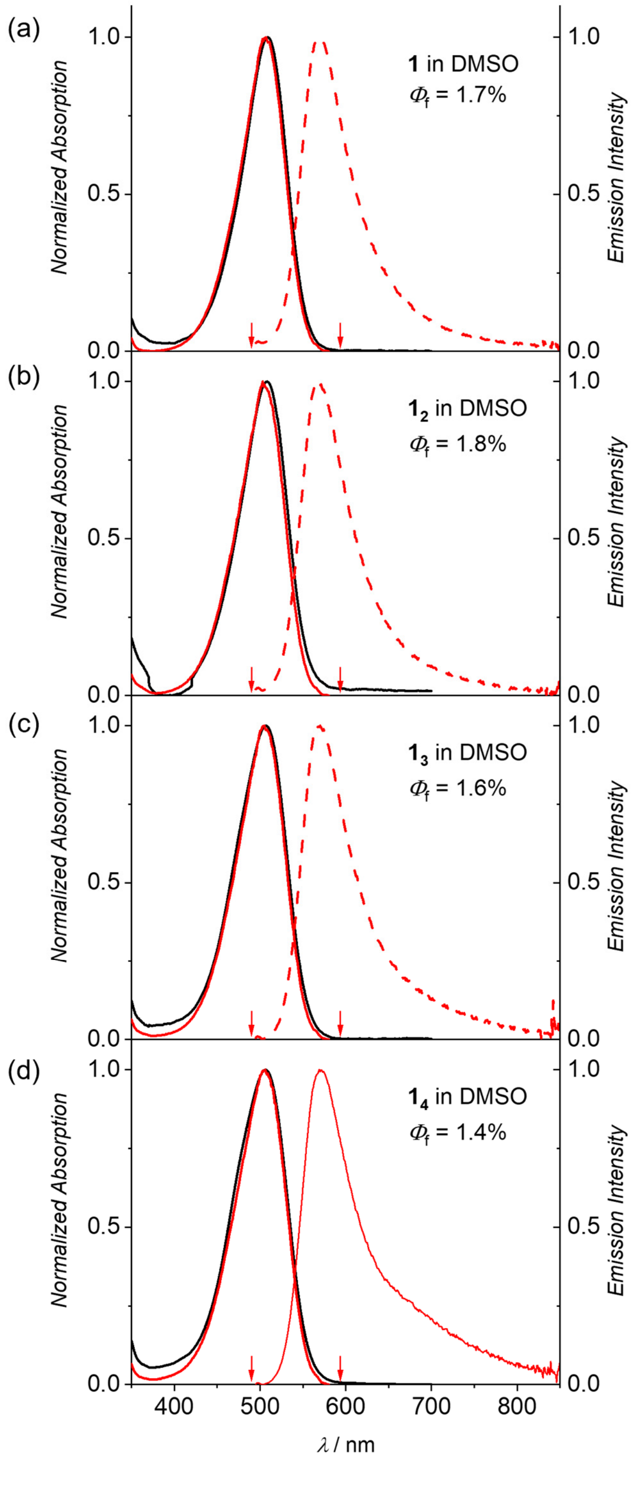

Fig. S8. Normalized UV-vis (in black), emission (in red, dashed) and excitation (in red, solid) spectra of the monomeric species of (a) **1**, (b) $\mathbf{1}_2$, (c) $\mathbf{1}_3$ and (d) $\mathbf{1}_4$ obtained at low concentrations (OD ≤ 0.05) in DMSO at 293 K. The excitation and emission wavelengths are indicated by arrows in the respective colour. Quantum yield was determined relative using *N,N'*-bis(2,6-diisopropylphenyl)-perylene-3,4:9,10-bis(dicarboximide) (100% in $CHCl_3$)[10] as a standard.

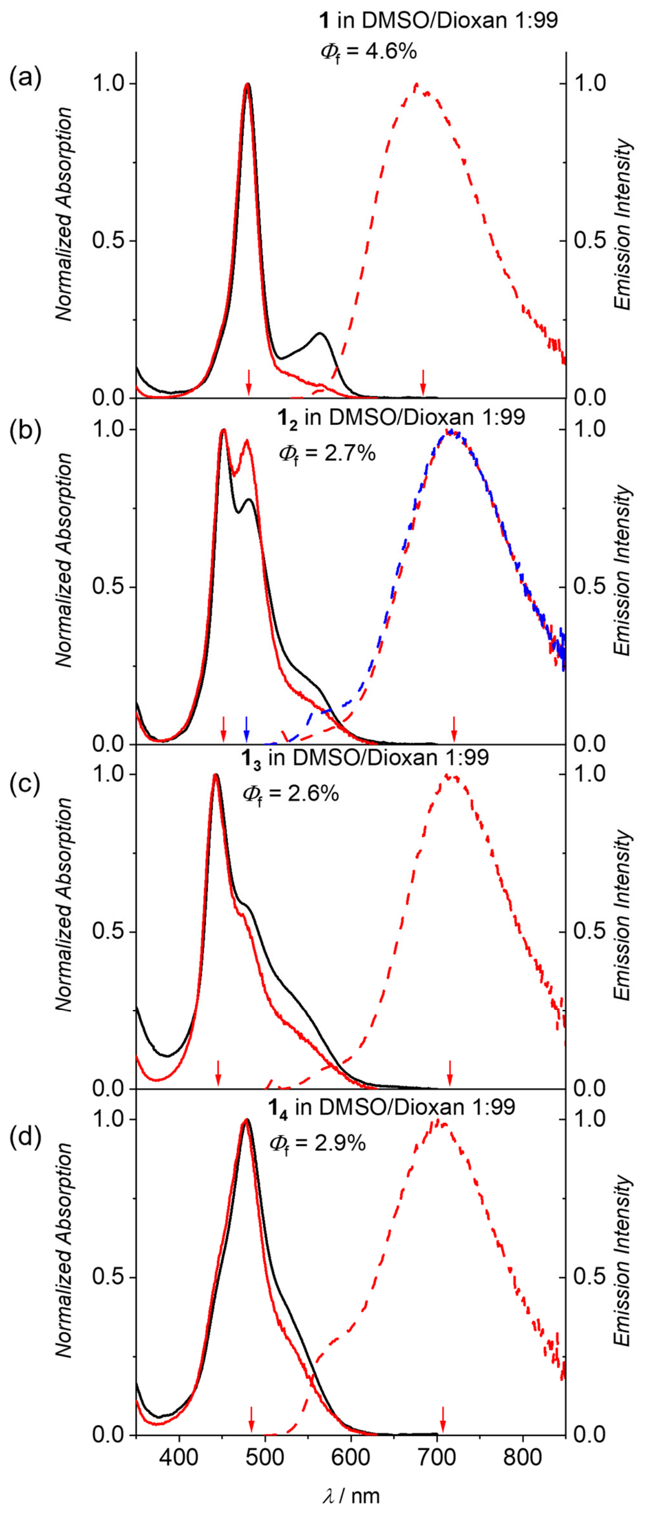

Fig. S9. Normalized UV-vis (in black), emission (in red and blue, dashed) and excitation (in red, solid) spectra of the predominantly dimerized species of (a) **1**, (b) $\mathbf{1_2}$, and (c) $\mathbf{1_3}$ as well as (d) aggregated/folded $\mathbf{1_4}$ obtained at low concentrations (OD ≤ 0.05) in DMSO/dioxane 1:99 at 293 K. The excitation and emission wavelengths are indicated by arrows in the respective colour. Quantum yield was determined relative using *N,N'*-bis(2,6-diisopropylphenyl)-1,6,7,12-tetraphenoxy-perylene-3,4:9,10-bis(dicarboximide) (96% in $CHCl_3$)[10] as a standard.

4. AFM analysis

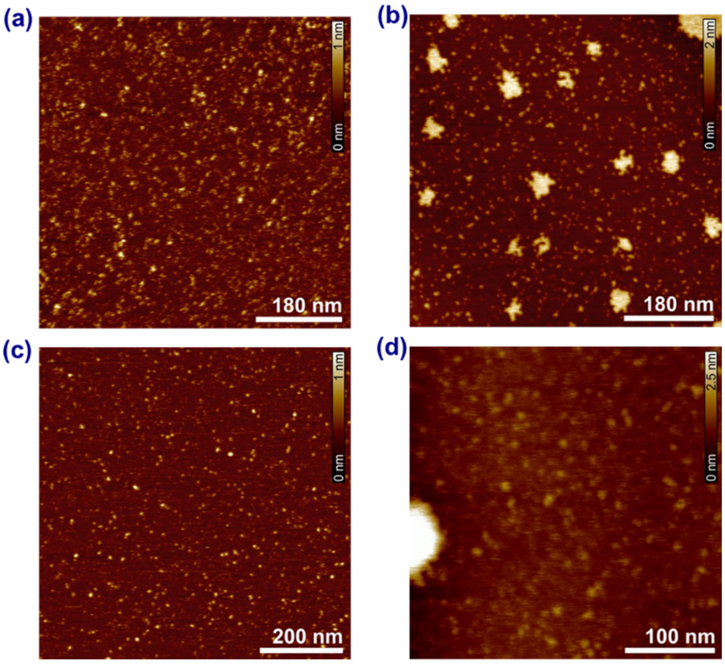

Fig. S10. Height AFM images of thin films of (a) **1**, (b) $\mathbf{1_2}$, (c) $\mathbf{1_3}$ and (d) $\mathbf{1_4}$ prepared by spin coating from a solution of the respective dye in 1% dmso/dioxane on mica ([**1**] = 3.2 x 10^{-6} M, [$\mathbf{1_2}$] = 1.8 x 10^{-6} M, [$\mathbf{1_3}$] = 1.2 x 10^{-6} M, [$\mathbf{1_4}$] = 0.92 x 10^{-6} M, 4000 rpm) showing layers composed of small spherical nanoparticles with a diameter of 0.37-0.50 nm $(\mathbf{1})_2$, 0.4-0.6 nm $(\mathbf{1_2})_2$, 0.35-0.50 nm $(\mathbf{1_3})_2$ and 0.40-0.60 nm $\mathbf{1_4}$ aggregates.

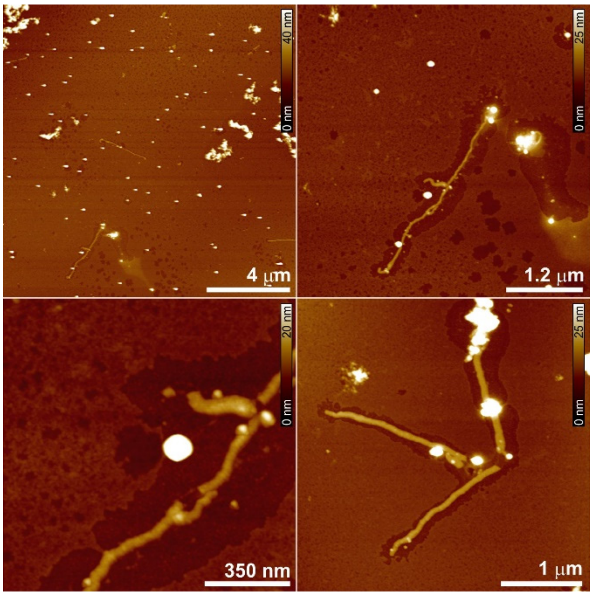

Fig. S11. Height AFM images of sample $\mathbf{1_4}$ in 20% $CHCl_3$/MCH (3.41 x 10^{-6} M). The height of the aggregates was measured to be 5.5-6.0 nm, while the lateral measured diameter varies between 25-60 nm.

5. DOSY NMR analysis

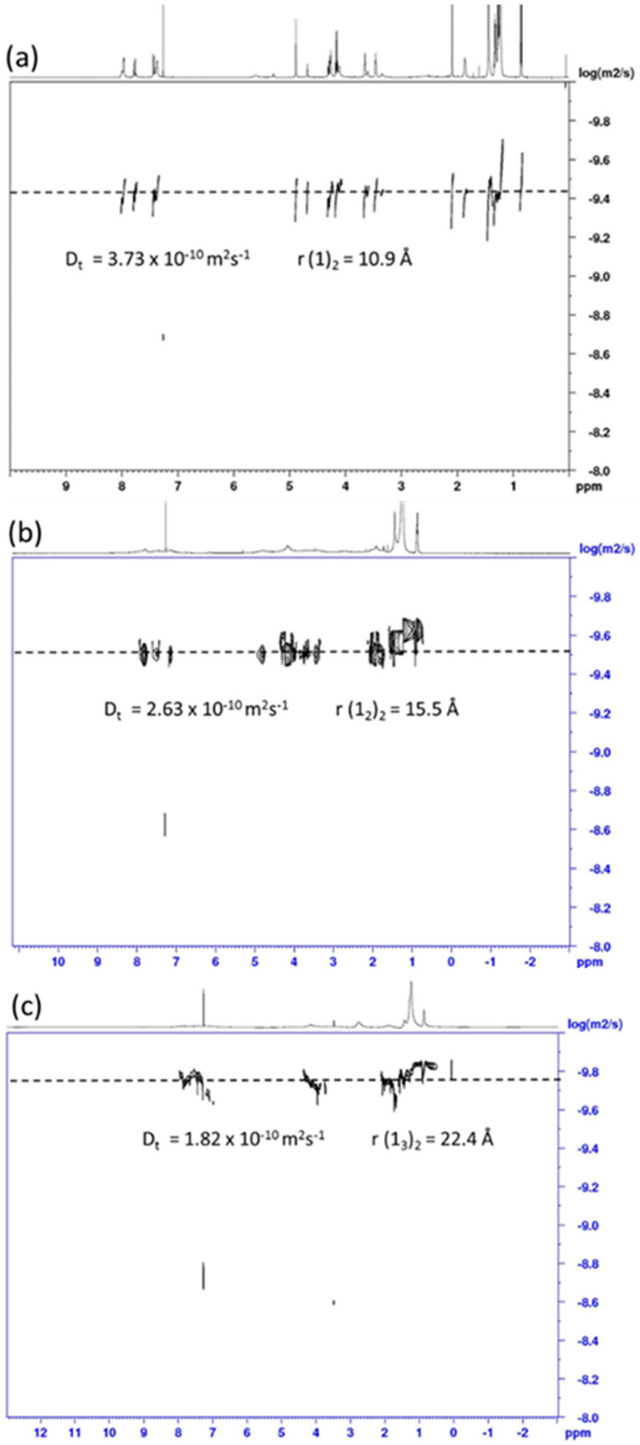

Fig. S12. ^{1}H DOSY NMR spectra (600 MHz) of the self-assembled structures (a) (**1**)$_2$, (b) (**1$_2$**)$_2$ and (c) (**1$_3$**)$_2$ in $CDCl_3$ at 295 K.

6. NMR spectra

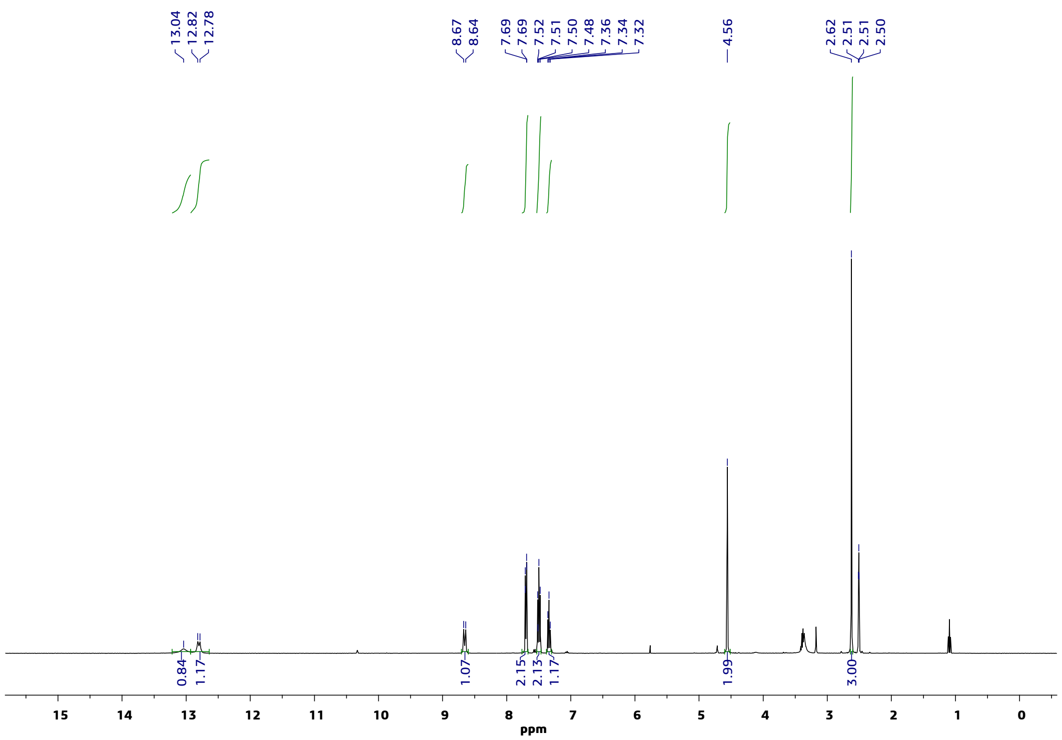

Fig. S13. ^{1}H NMR spectrum (400 MHz) of **3** in DMSO at 295 K.

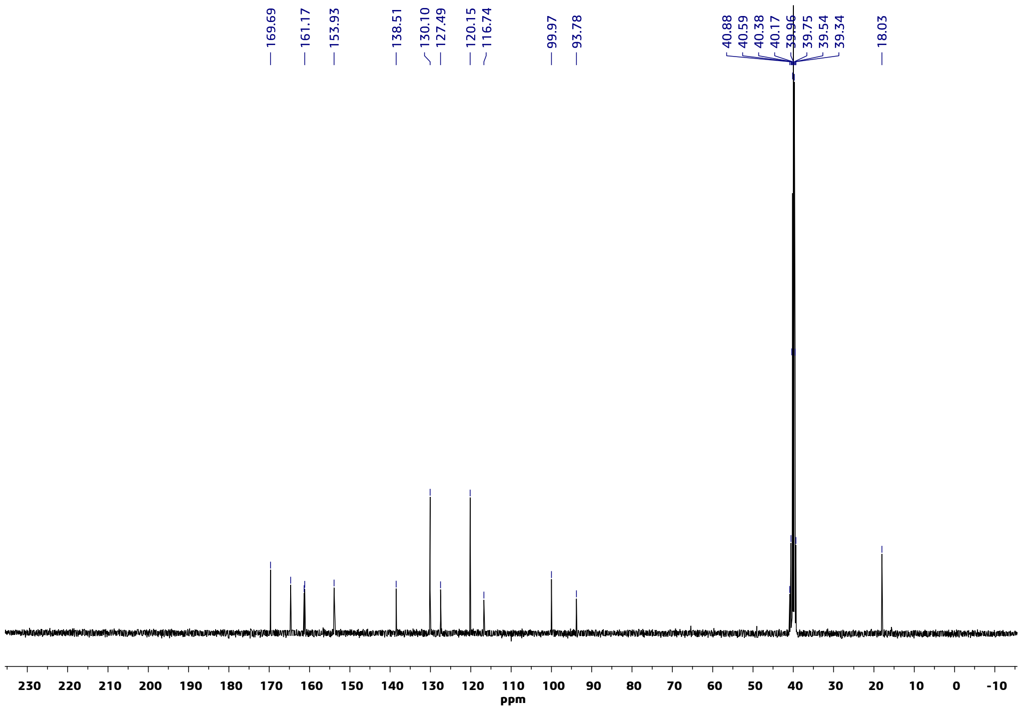

Fig. S14. ^{13}C NMR spectrum (101 MHz) of **3** in DMSO at 295 K.

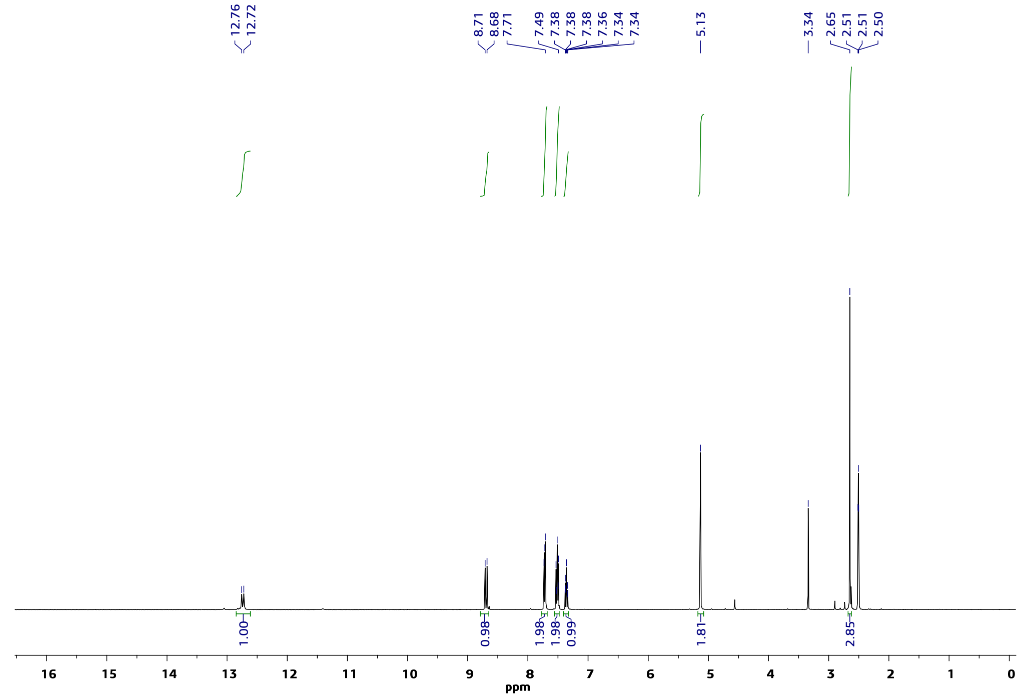

Fig. S15. ^{1}H NMR spectrum (400 MHz) of **4** in DMSO at 295 K.

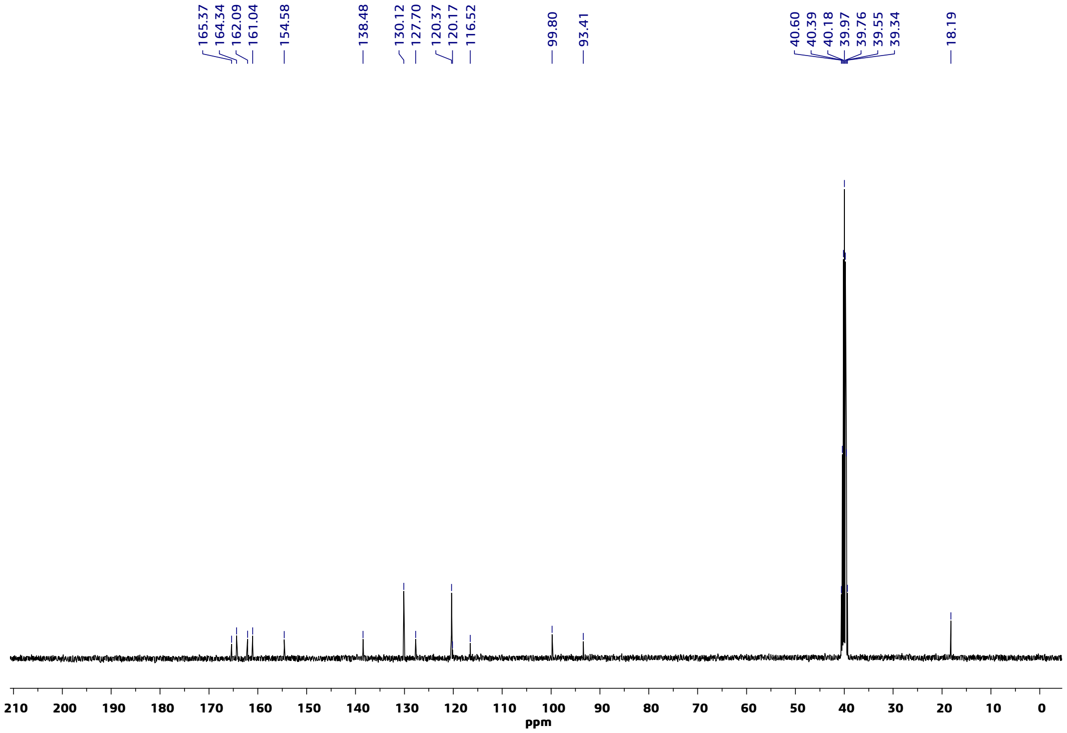

Fig. S16. ^{13}C NMR spectrum (101 MHz) of **4** in DMSO at 295 K.

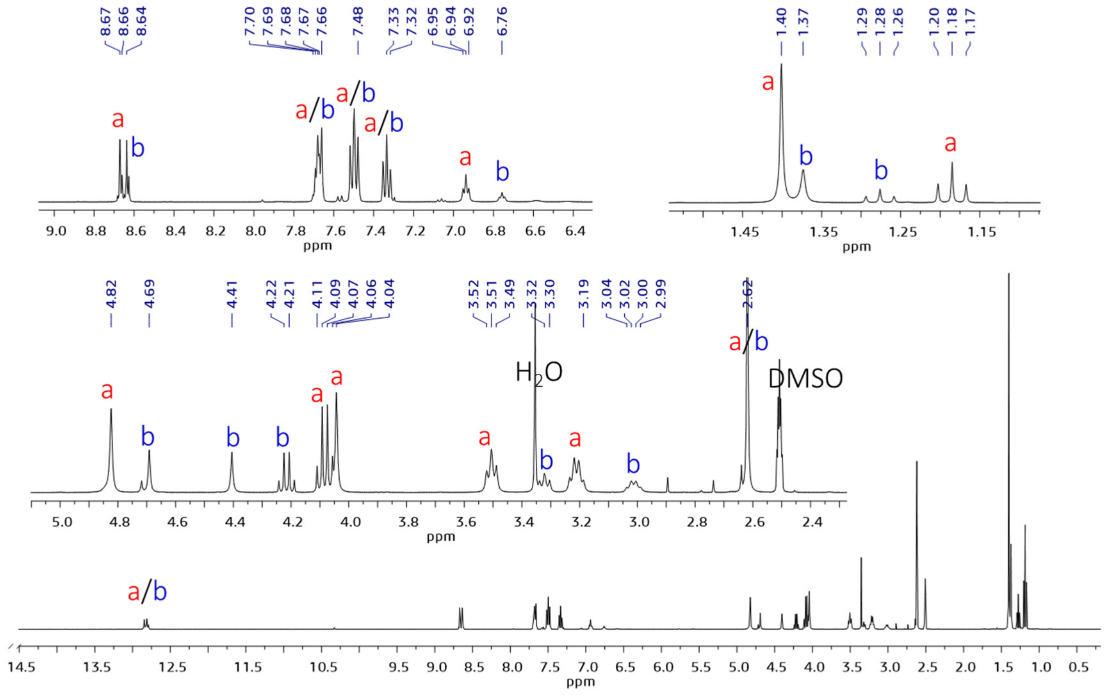

Fig. S17. ^{1}H NMR spectrum (400 MHz) of **5** in DMSO at 295 K.

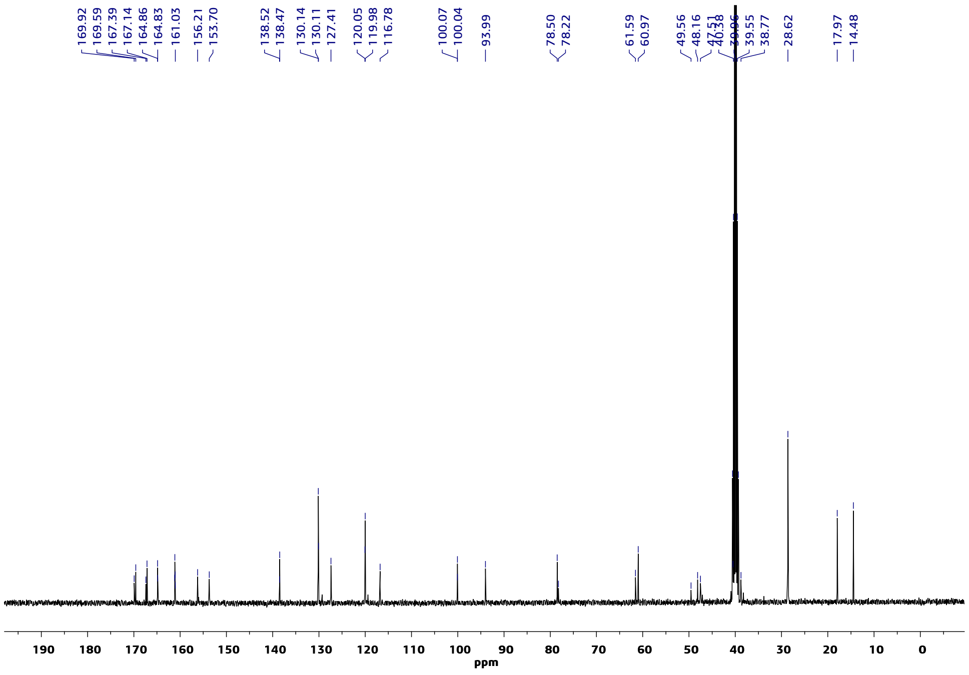

Fig. S18. ^{13}C NMR spectrum (101 MHz) of **5** in DMSO at 295 K.

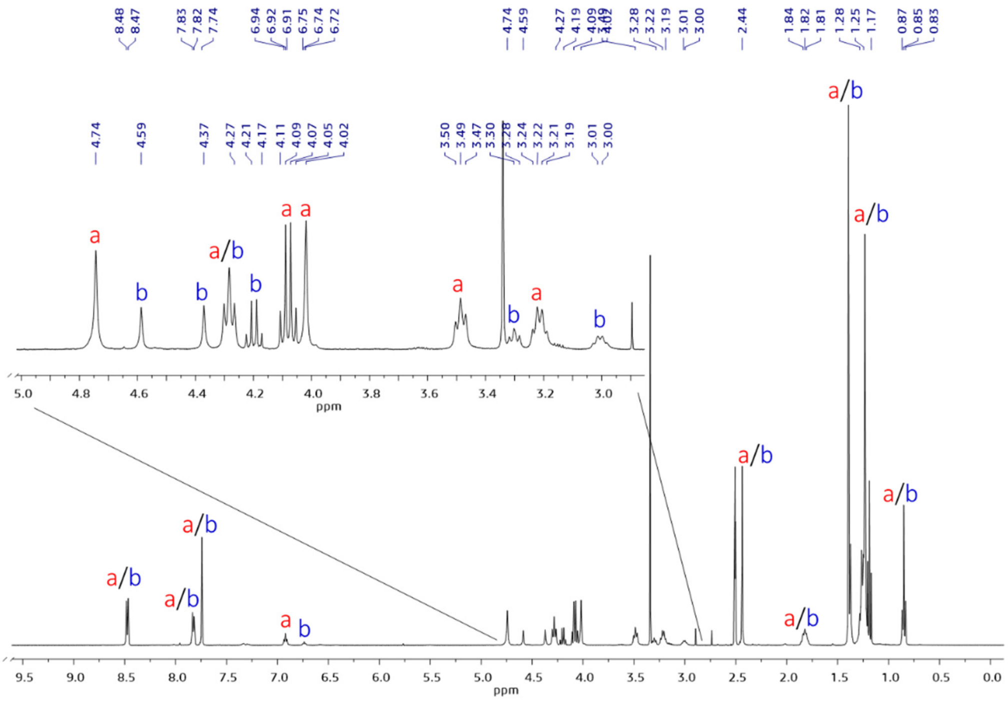

Fig. S19. ^{1}H NMR spectrum (400 MHz) of **1** in DMSO at 295 K.

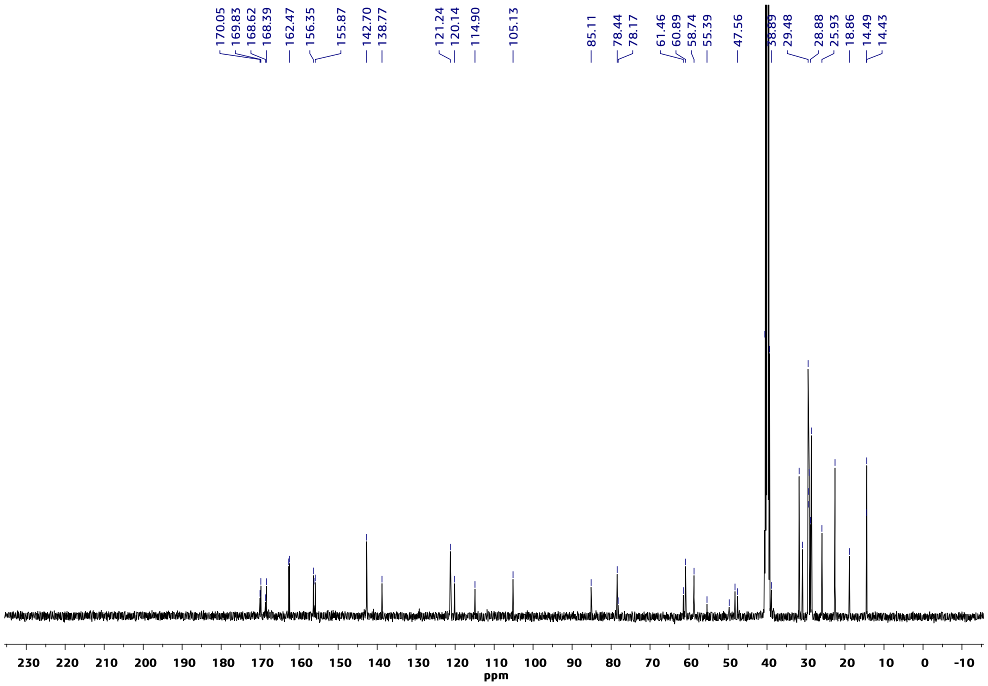

Fig. S20. ^{13}C NMR spectrum (101 MHz) of **1** in DMSO at 295 K.

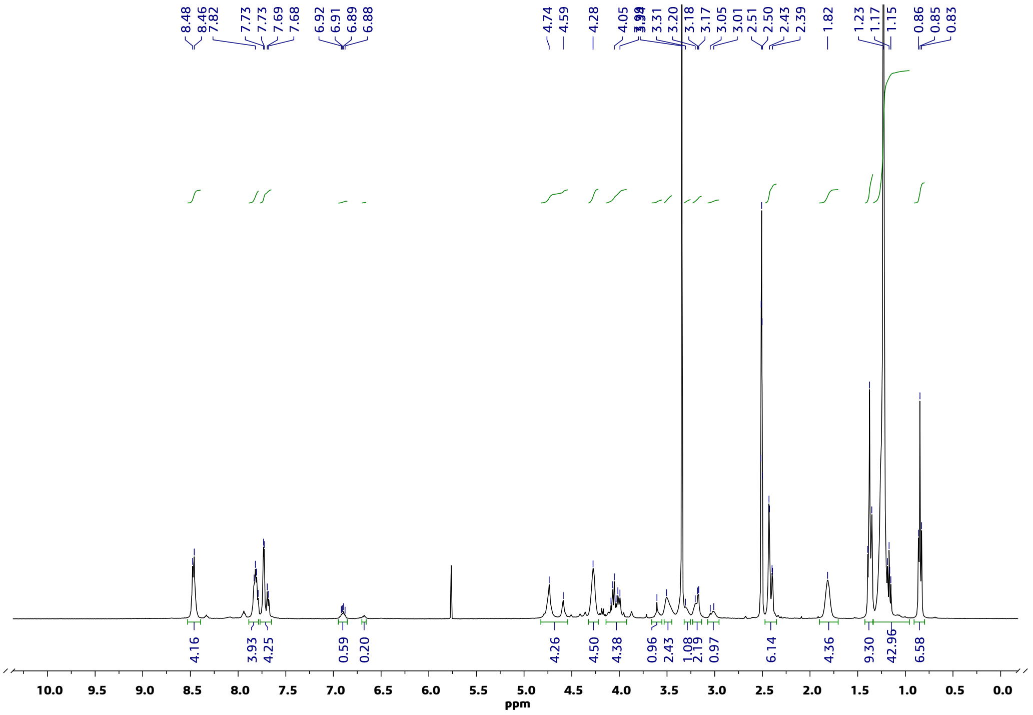

Fig. S21. ^{1}H NMR spectrum (400 MHz) of $\mathbf{1_2}$ in DMSO at 295 K.

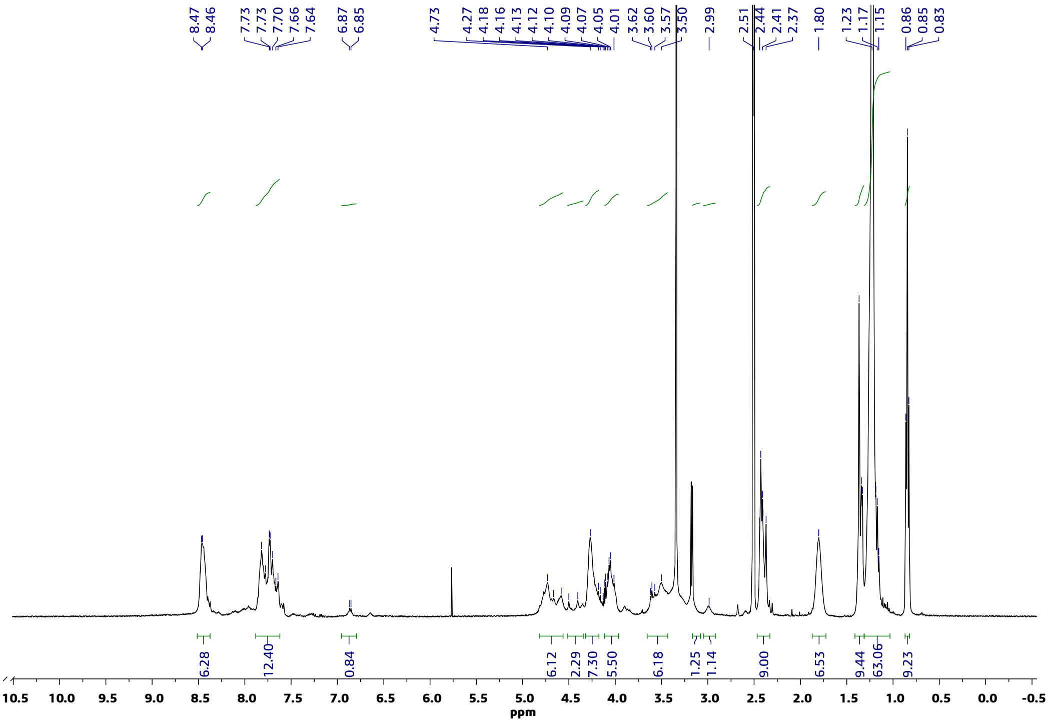

Fig. S22. ^{1}H NMR spectrum (400 MHz) of $\mathbf{1_3}$ in DMSO at 295 K.

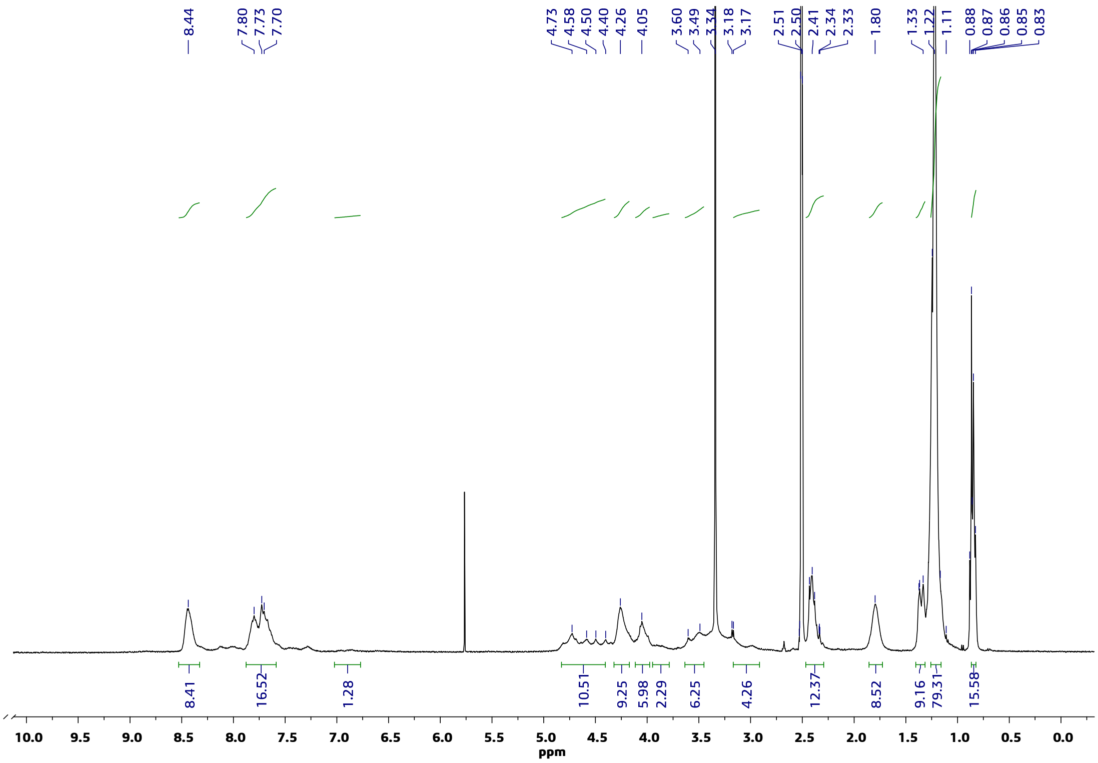

Fig. S23. ^{1}H NMR spectrum (400 MHz) of $\mathbf{1_4}$ in DMSO at 295 K.

7. MS spectra

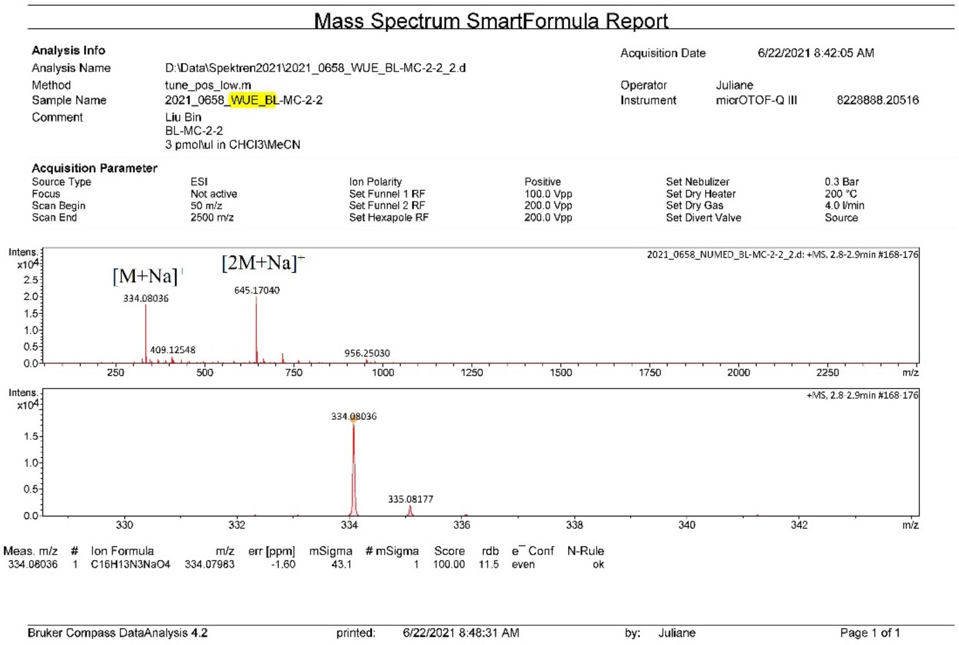
Mass Spectrum SmartFormula Report

Analysis Info

Analysis Name	D:\Data\Spektren2021\2021_0658_WUE_BL-MC-2-2_2.d	Acquisition Date	6/22/2021 8:42:05 AM
Method	tune_pos_low.m	Operator	Juliane
Sample Name	2021_0658_WUE_BL-MC-2-2	Instrument	micrOTOF-Q III 8228888.20516
Comment	Liu Bin<br>BL-MC-2-2<br>3 pmol\ul in CHCl3\MeCN		

Acquisition Parameter

Source Type	ESI	Ion Polarity	Positive	Set Nebulizer	0.3 Bar
Focus	Not active	Set Funnel 1 RF	100.0 Vpp	Set Dry Heater	200 °C
Scan Begin	50 m/z	Set Funnel 2 RF	200.0 Vpp	Set Dry Gas	4.0 l/min
Scan End	2500 m/z	Set Hexapole RF	200.0 Vpp	Set Divert Valve	Source

Meas. m/z	#	Ion Formula	m/z	err [ppm]	mSigma	# mSigma	Score	rdb	e⁻ Conf	N-Rule
334.08036	1	C16H13N3NaO4	334.07983	-1.60	43.1	1	100.00	11.5	even	ok

Bruker Compass DataAnalysis 4.2 printed: 6/22/2021 8:48:31 AM by: Juliane Page 1 of 1

Fig. S24. MS spectrum of **3**

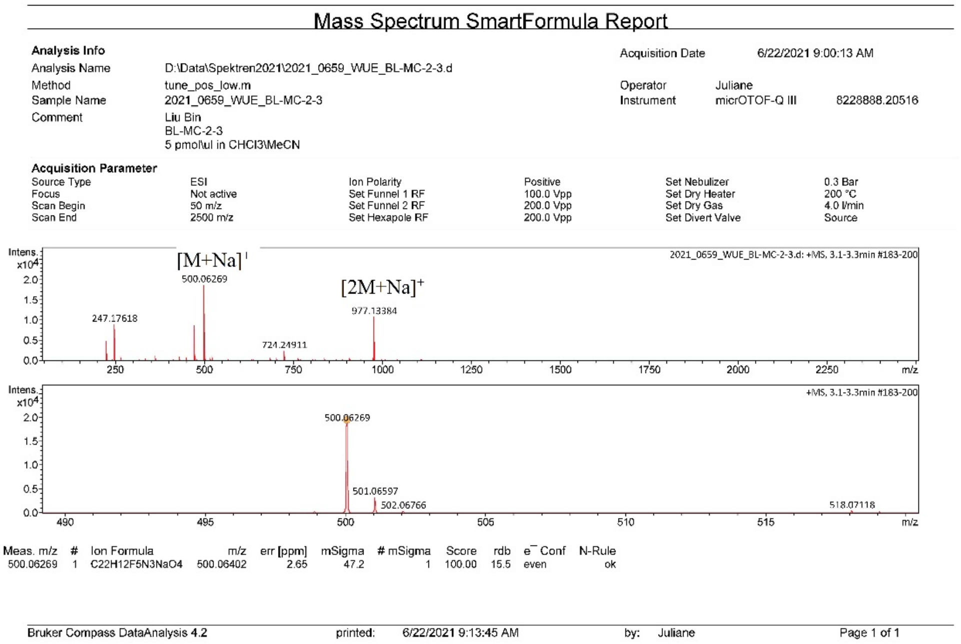
Mass Spectrum SmartFormula Report

Analysis Info

Analysis Name	D:\Data\Spektren2021\2021_0659_WUE_BL-MC-2-3.d	Acquisition Date	6/22/2021 9:00:13 AM
Method	tune_pos_low.m	Operator	Juliane
Sample Name	2021_0659_WUE_BL-MC-2-3	Instrument	micrOTOF-Q III 8228888.20516
Comment	Liu Bin<br>BL-MC-2-3<br>5 pmol\ul in CHCl3\MeCN		

Acquisition Parameter

Source Type	ESI	Ion Polarity	Positive	Set Nebulizer	0.3 Bar
Focus	Not active	Set Funnel 1 RF	100.0 Vpp	Set Dry Heater	200 °C
Scan Begin	50 m/z	Set Funnel 2 RF	200.0 Vpp	Set Dry Gas	4.0 l/min
Scan End	2500 m/z	Set Hexapole RF	200.0 Vpp	Set Divert Valve	Source

Meas. m/z	#	Ion Formula	m/z	err [ppm]	mSigma	# mSigma	Score	rdb	e⁻ Conf	N-Rule
500.06269	1	C22H12F5N3NaO4	500.06402	2.65	47.2	1	100.00	15.5	even	ok

Bruker Compass DataAnalysis 4.2 printed: 6/22/2021 9:13:45 AM by: Juliane Page 1 of 1

Fig. S25. MS spectrum of **4**

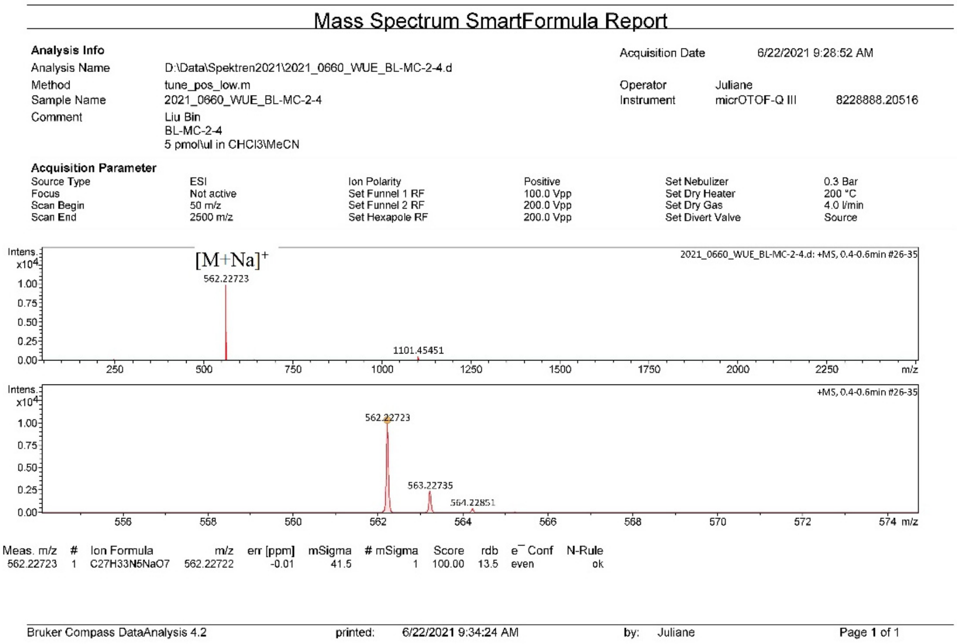

Fig. S26. MS spectrum of **5**

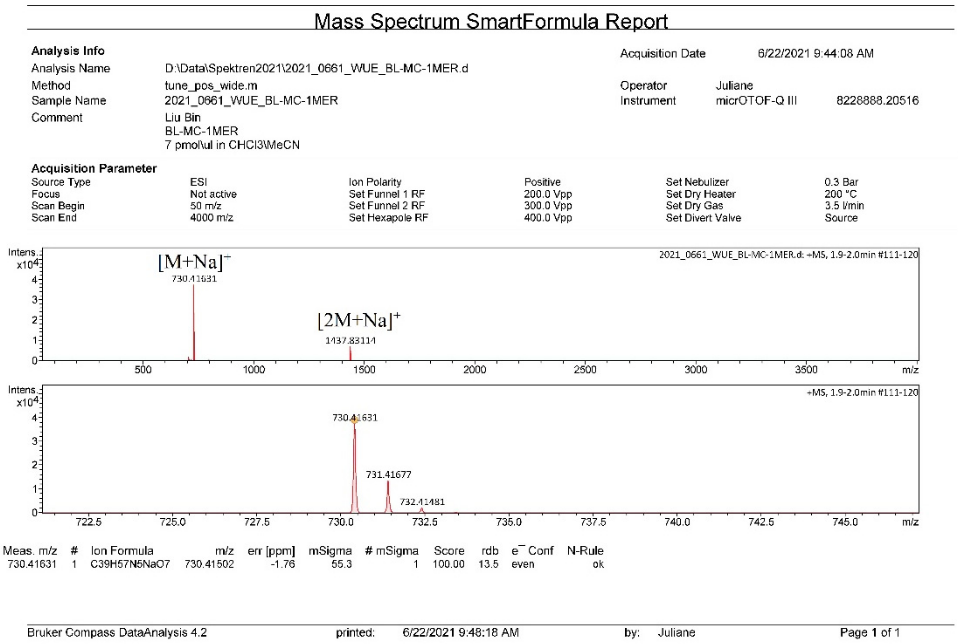

Fig. S27. MS spectrum of **1**

Mass Spectrum SmartFormula Report

Analysis Info		Acquisition Date	6/22/2021 9:55:15 AM	
Analysis Name	D:\Data\Spektren2021\2021_0662_WUE_BL-MC-2MER.d			
Method	tune_pos_high.m	Operator	Juliane	
Sample Name	2021_0662_WUE_BL-MC-2MER	Instrument	micrOTOF-Q III	8228888.20516
Comment	Liu Bin<br>BL-MC-2MER<br>13 pmol\ul in CHCl3\MeCN			

Acquisition Parameter					
Source Type	ESI	Ion Polarity	Positive	Set Nebulizer	0.3 Bar
Focus	Not active	Set Funnel 1 RF	400.0 Vpp	Set Dry Heater	200 °C
Scan Begin	600 m/z	Set Funnel 2 RF	600.0 Vpp	Set Dry Gas	3.5 l/min
Scan End	3000 m/z	Set Hexapole RF	800.0 Vpp	Set Divert Valve	Source

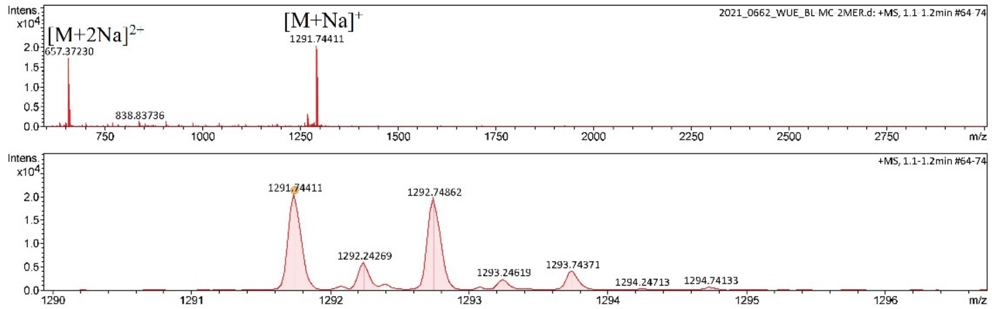

Meas. m/z	#	Ion Formula	m/z	err [ppm]	mSigma	# mSigma	Score	rdb	e⁻ Conf	N-Rule
1291.74411	1	C71H100N10NaO11	1291.74652	1.87	98.0	1	100.00	26.5	even	ok

Bruker Compass DataAnalysis 4.2 printed: 6/22/2021 10:02:37 AM by: Juliane Page 1 of 1

Fig. S28. MS spectrum of $\mathbf{1_2}$

Mass Spectrum SmartFormula Report

Analysis Info		Acquisition Date	6/22/2021 10:12:24 AM	
Analysis Name	D:\Data\Spektren2021\2021_0663_WUE_BL-MC-3MER.d			
Method	tune_pos_high.m	Operator	Juliane	
Sample Name	2021_0663_WUE_BL-MC-3MER	Instrument	micrOTOF-Q III	8228888.20516
Comment	Liu Bin<br>BL-MC-3MER<br>18 pmol\ul in CHCl3\MeCN			

Acquisition Parameter					
Source Type	ESI	Ion Polarity	Positive	Set Nebulizer	0.3 Bar
Focus	Not active	Set Funnel 1 RF	400.0 Vpp	Set Dry Heater	200 °C
Scan Begin	600 m/z	Set Funnel 2 RF	600.0 Vpp	Set Dry Gas	3.5 l/min
Scan End	3000 m/z	Set Hexapole RF	800.0 Vpp	Set Divert Valve	Source

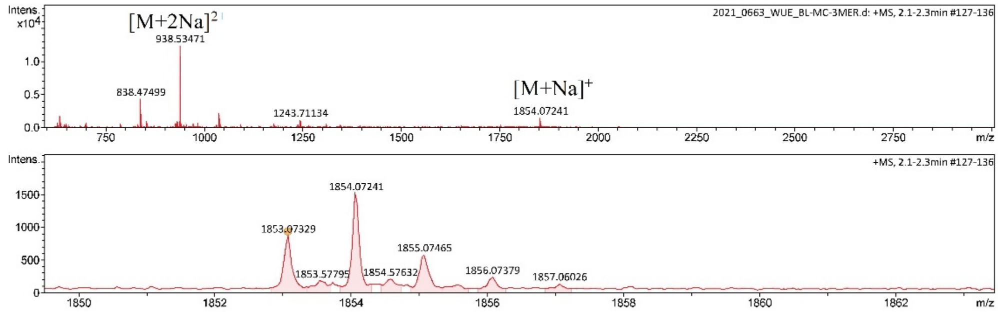

Meas. m/z	#	Ion Formula	m/z	err [ppm]	mSigma	# mSigma	Score	rdb	e⁻ Conf	N-Rule
1853.07329	1	C103H143N15NaO15	1853.07803	2.56	149.3	1	100.00	39.5	even	ok

Bruker Compass DataAnalysis 4.2 printed: 6/22/2021 10:18:10 AM by: Juliane Page 1 of 1

Fig. S29. MS spectrum of $\mathbf{1_3}$

Mass Spectrum SmartFormula Report

Analysis Info

Analysis Name	D:\Data\Spektren2022\2022_0080_WUE_BL-MC.d	Acquisition Date	2/4/2022 11:47:21 AM
Method	tune_pos_high.m	Operator	Juliane
Sample Name	2022_0080_WUE_BL-MC	Instrument	micrOTOF-Q III 8228888.20516
Comment	Vonhausen Yvonne<br>BL-MC-ABBA<br>24 pmol\ul in MeCN\CHCl3		

Acquisition Parameter

Source Type	ESI	Ion Polarity	Positive	Set Nebulizer	0.3 Bar
Focus	Not active	Set Funnel 1 RF	400.0 Vpp	Set Dry Heater	200 °C
Scan Begin	600 m/z	Set Funnel 2 RF	600.0 Vpp	Set Dry Gas	3.5 l/min
Scan End	3000 m/z	Set Hexapole RF	800.0 Vpp	Set Divert Valve	Source

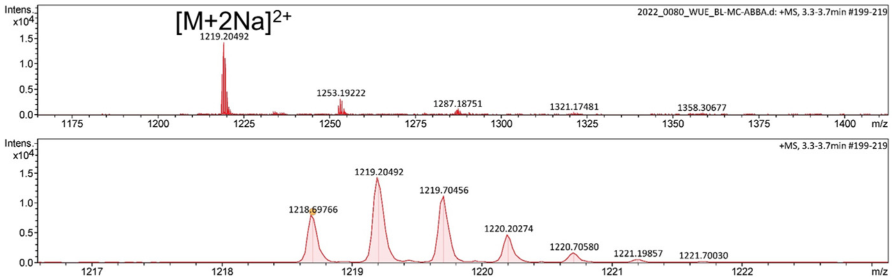

Meas. m/z	#	Ion Formula	m/z	err [ppm]	mSigma	# mSigma	Score	rdb	e⁻ Conf	N-Rule
1218.69766	1	C135H186N20Na2O19	1218.69938	1.41	58.6	1	100.00	52.0	even	ok

Bruker Compass DataAnalysis 4.2 printed: 2/4/2022 11:53:25 AM by: Juliane Page 1 of 1

Fig. S30. MS spectrum of $\mathbf{1_4}$

References

[1] R. Ovadia, A. Lebrun, I. Barvik, J. J. Vasseur, C. Baraguey, K. Alvarez, *Org. Biomol. Chem.* **2015**, *13*, 11052-11071.

[2] P. Clivio, D. Guillaume, M. T. Adeline, J. Hamon, C. Riche, J. L. Fourrey, *J. Am. Chem. Soc.* **1998**, *120*, 1157-1166.

[3] J. Lađarević, B. Božić, L. Matović, B. B. Nedeljković, D. Mijin, *Dyes Pigm.* **2019**, *162*, 562-572.

[4] J. C. Yu, Y. J. Cui, C. D. Wu, Y. Yang, Z. Y. Wang, M. O'Keeffe, B. L. Chen, G. D. Qian, *Angew. Chem.* **2012**, *124*, 10694-10697; *Angew. Chem. Int. Edit.* **2012**, *51*, 10542-10545.

[5] M. Komiyama, Y. Aiba, T. Ishizuka, J. Sumaoka, *Nat. Protoc.* **2008**, *3*, 646-654.

[6] E. Kirchner, D. Bialas, F. Fennel, M. Grune, F. Würthner, *J. Am. Chem. Soc.* **2019**, *141*, 7428-7438.

[7] F. Fennel, S. Wolter, Z. Q. Xie, P. A. Plotz, O. Kuhn, F. Würthner, S. Lochbrunner, *J. Am. Chem. Soc.* **2013**, *135*, 18722-18725.

[8] J. Gershberg, F. Fennel, T. H. Rehm, S. Lochbrunner, F. Würthner, *Chem. Sci.* **2016**, *7*, 1729-1737.

[9] Z. Chen, A. Lohr, C. R. Saha-Möller, F. Würthner, *Chem. Soc. Rev.* **2009**, *38*, 564.

[10] G. Seybold, G. Wagenblast, *Dyes Pigm.* **1989**, *11*, 303–317.
